# NOR1 promotes the osteoblastic differentiation of human periodontal ligament stem cells via TGF-β signaling pathway

**DOI:** 10.1007/s00018-024-05356-3

**Published:** 2024-08-09

**Authors:** Yun Wu, Huan Jing, Yicun Li, Mengqing Li, Yating Zheng, Yuntao Lin, Guixing Ma, Huiling Cao, Hongyu Yang

**Affiliations:** 1 The Institute of Stomatology, Peking University Shenzhen Hospital, Shenzhen Peking University-The Hong Kong University of Science and Technology Medical Center, Shenzhen, Guangdong 518000 China; 2 Guangdong Provincial High-level Clinical Key Specialty, Shenzhen, Guangdong 518000 China; 3 Guangdong Province Engineering Research Center of Oral Disease Diagnosis and Treatment, Shenzhen, Guangdong 518000 China; 4 Department of Oral and Maxillofacial Surgery, Stomatological Center, Peking University Shenzhen Hospital, Shenzhen, Guangdong 518000 China; 5 Department of Pathology, Peking University Shenzhen Hospital, Shenzhen, Guangdong 518000 China; 6 Department of Biochemistry, School of Medicine, Southern University of Science and Technology, Key University Laboratory of Metabolism and Health of Guangdong, Guangdong Provincial Key Laboratory of Cell Microenvironment and Disease Research, Southern University of Science and Technology, Shenzhen, Guangdong 518055 China

**Keywords:** Periodontal ligament stem cell (PDLSC), Osteogenic differentiation, Neuron-derived orphan receptor 1 (NOR1), Transforming growth factor beta receptor 1 (TGFBR1), TGF-β signaling pathway

## Abstract

Alveolar bone loss is a main manifestation of periodontitis. Human periodontal ligament stem cells (PDLSCs) are considered as optimal seed cells for alveolar bone regeneration due to its mesenchymal stem cell like properties. Osteogenic potential is the premise for PDLSCs to repair alveolar bone loss. However, the mechanism regulating osteogenic differentiation of PDLSCs remain elusive. In this study, we identified Neuron-derived orphan receptor 1 (NOR1), was particularly expressed in PDL tissue in vivo and gradually increased during osteogenic differentiation of PDLSCs in vitro. Knockdown of NOR1 in hPDLSCs inhibited their osteogenic potential while NOR1 overexpression reversed this effect. In order to elucidate the downstream regulatory network of NOR1, RNA-sequencing was used. We found that downregulated genes were mainly enriched in TGF-β, Hippo, Wnt signaling pathway. Further, by western blot analysis, we verified that the expression level of phosphorylated-SMAD2/3 and phosphorylated-SMAD4 were all decreased after NOR1 knockdown. Additionally, ChIP-qPCR and dual luciferase reporter assay indicated that NOR1 could bind to the promoter of TGFBR1 and regulate its activity. Moreover, overexpression of TGFBR1 in PDLSCs could rescue the damaged osteogenic potential after NOR1 knockdown. Taken together, our results demonstrated that NOR1 could activate TGF-β/SMAD signaling pathway and positively regulates the commitment of osteoblast lineages of PDLSCs by targeting TGFBR1 directly.

**Supplementary Information:**

The online version contains supplementary material available at 10.1007/s00018-024-05356-3.

## Introduction

Alveolar bone is the main structure of periodontal supporting tissue, playing an important role in tooth development, eruption and chewing movement [1]. Alveolar bone loss is usually caused by periodontitis, trauma, tumor or genetic defects. Regeneration of the alveolar bone remains a challenge for dentist and researchers. At present, many researchers have tried to regenerate the alveolar bone based on tissue engineering technology which involves stem cells, biological signals, and cell-seeded scaffold [2]. With the biological characteristics of clonal proliferation, immune regulation and multi-lineage differentiation potential, periodontal ligament stem cells (PDLSCs) were considered as the optimal seed cells for alveolar bone regeneration [3]. In a clinical trial, researchers used human PDLSCs derived from wisdom teeth to generate cell sheets and transplanted them with a β-TCP scaffold and biodegradable polyglycolic acid mesh into the periodontal defects. At 6 months of post‐surgery, all patients showed a increase of bone height [4].

During alveolar bone regeneration, PDLSCs undergone osteogenic differentiation. In order to improve the efficiency of PDLSCs in repairing periodontal bone defects, it is imperative to clarify the molecular mechanisms underlying the osteogenic differentiation of PDLSCs. Previous studies have indicated that transcription factors, such as RUNX2, SP7, MSX2, DLX5 and ATF4 [5–8], play an vital role in initiating and modulating the osteoblast differentiation of PDLSCs by binding to cis-regulatory elements such as promoters and enhancers. However, the transcription factor network governing the osteogenic process of PDLSCs has not yet been fully explored.

Neuron-derived orphan receptor 1 (NOR1), a member of orphan nuclear receptor family that includes Nur77 and Nurr1 [9], is widely expressed [10–13]. Functionally, NOR1 is a transcription factor involved in adipogenesis, inflammation, vascular remodeling, glucose and lipid metabolism [14, 15]. For Nurr1 and Nur77, their role in bone homeostasis has been widely studied [16, 17]. It was reported that reduced Nurr1 expression in MC3T3-E1 cells and primary cultured mouse calvarial osteoblasts decreased the expression of osteoblast differentiation marker genes, including osteocalcin (OCN) and collagen type I alpha 1(COL1A1) [16]. Another research revealed that myeloid specific deletion of Nur77 resulted in osteopenia [17]. NOR1 shares highly homology with Nurr1 and Nur77, suggesting they may possess similar functions. More importantly, it has been reported that the expression of NOR1 was markedly upregulated in parathyroid hormone (PTH) stimulated mouse skulls and long bone-derived osteoblasts [18], which indicates that NOR1 may be involved in the process of osteogenic differentiation. However, the specific role of NOR1 in osteogenic differentiation and bone formation has not yet been reported.

In the present study, we explored the expression of NOR1 in PDL tissue in vivo and during osteoblast differentiation of PDLSCs in vitro. Additionally, we elucidated the effects of NOR1 on the commitment of osteoblast lineages of PDLSCs by gain-of-function and loss-of-function assays. Combining RNA sequencing (RNA-seq) and ChIP sequencing (ChIP-seq) analysis, we revealed the genomewide profile of NOR1-regulated genes and downstream signaling pathways during osteoblast differentiation of PDLSCs.

## Materials and methods

### Cell culture

Human PDLSCs were isolated from the donor who experienced tooth extraction for orthodontic reason at the department of stomatology, Peking University Shenzhen Hospital. This study was approved by the ethics committee of Peking University Shenzhen Hospital (Approval Number: 2024-032-01). The informed consent was obtained from the donors. In brief, the teeth were washed in sterilized PBS until the roots turned white. The periodontal ligament tissue was scraped from the middle third of tooth root, carefully. Subsequently, the tissue was digested with 3 mg/ml collagenase and 4 mg/ml dispase enzyme. After 1 h, the digestion solution was centrifugated, the supernatant was discarded and the cell sediment was re-suspended in alpha MEM medium supplemented with 10% fetal bovine serum, 1% penicilin and streptomycin. The cell culture medium was changed every two day. The passage 3–6 of PDLSCs were used in the subsequent experiments.

### Osteogenic differentiation

PDLSCs were seeded on 6 well plate with the density of 1 × 10^5^ cells/well. After reaching sub-confluence, cells were treated with osteogenic cocktail purchased from Cyagen Biosciences Inc. (Guangzhou, China) according to the manufacturer’s instruction. After osteogenic induction, cells were collected for subsequent experiments.

### Quantitative reverse transcriptase-polymerase chain reaction (RT-qPCR) analysis

Total RNA was extracted from PDLSCs using TRIzol reagent (Invitrogen, Carlsbad, CA, USA). Then, a total of one µg RNA was reverse transcribed into cDNA using the PrimeScript™ RT Master Mix kit (Takara, Japan) according to manufacturer’ s instruction. Subsequently, cDNA was used as the template for qPCR analysis using SYBR Premix EX Taq solution (Takara, Japan) according to the manufacturer’s protocol. The thermocycle conditions used in amplification were as follows: 95°C for 5 s; 60 °C for 30 s; 72 °C for 30 s. The primers used in this experiment are listed in Table 1. β-actin was used as the internal control. Data analysis was performed using the 2^−ΔΔCq^ method reported by Livak and Schmittgen [19]. All qPCR assays were repeated at least three times.

**Table 1:** Primer sequences for RT-qPCR

Gene	Forward primer(5’-3’)	Reverse primer(5’-3’)
ALPL	AAGTACTGGCGAGACCAAGC	CACTGTGGAGACACCCATCC
RUNX2	TAGGCGCATTTCAGGTGCTT	GGACATACCGAGGGACATGC
COL1A1	AGTGGTTTGGATGGTGCCAA	GCACCATCATTTCCACGAGC
OCN	ATGAGAGCCCTCACACTCCT	CTTGGACACAAAGGCTGCAC
NOR1	GACCTTGGCAGCACTGAGAT	TACACGCAGGAAGGCTTGAG
TGFBR1	CCTCGAGATAGGCCGTTTGT	GCAATGGTAAACCAGTAGTTGGA
β-actin	CCGCGAGAAGATGACCCAG	GATAGCACAGCCTGGATAGCA

### Western-blot analysis

Cells in each group were harvested and lysed with RIPA buffer supplemented with protease and phoshatase inhibitors (Roche, Germany). The cell lysate was centrifuged at 12,000 rpm for 10 min. The protein concentration was measured using a BCA protein assay kit (Thermo, USA). An equal amount of protein from each sample was loaded into SDS-PAGE gels for electrophoresis and then transferred onto PVDF membrane (Millipore, Billerica, MA, USA). The transferred membrane was blocked with QuickBlock™ Blocking buffer (Beyotime, Wuhan, China) for 20 min and incubated with primary antibodies overnight. The primary antibodies used were as follows: NOR1 (1:1000, Proteintech Group.Inc, Rosemont, USA), RUNX2 (1:1000, Cell Signaling Technology.Inc, Boston, USA), OCN(1:1000, Bioss, Beijing, China), COL1A1 (1:1000, Cell Signaling Technology.Inc, Boston, USA), TGFBR1 (1:1000, Invitrogen, California, USA), Phosphorylated-SMAD2/3 (1:1000, Cell Signaling Technology.Inc, Boston, USA), Phosphorylated-SMAD4 (1:1000, Abcepta, San Diego, USA), SMAD2/3 (1:1000, Cell Signaling Technology.Inc, Boston, USA), SMAD4 (1:1000, Cell Signaling Technology.Inc, Boston, USA), SMAD7(1:1000, Abcepta, San Diego, USA) and β-actin (1:5000, Bioss, Beijing, China). Subsequently, the membranes were incubated with horseradish peroxidase-conjugated secondary antibodies for 1 h at room temperature. The protein bands were visualized using an enhanced chemiluminescence (ECL) kit (Millipore, Billerica, MA, USA) and captured using Tanon 5200 chemiluminescent imaging system (shanghai, China). β-actin was used as the internal reference.

### ALP staining

PDLSCs were cultured on 12 well plate at a density of 4 × 10^3^ cells/well. After cell adhesion, the culture medium was replaced with osteogenic cocktail containing 50 µg/ml ascorbic acid, 10 mmol/L β-glycerophosphate, and 10 nmol/L dexamethasone. The osteogenic medium was replaced every two day. After induction for 7 d, PDLSCs were stained for ALP using a BCIP/NBT kit (Beyotime, China) according to the manufacturer’s instruction.

### Alizarin red staining

For Alizarin red staining, PDLSCs were seeded on 12 well plate at a density of 4 × 10^3^ cells/well. Cells were cultured in osteogenic medium. After 21d, cells were rinsed with PBS, fixed with 4% paraformaldehyde for 15 min. And next, cells were stained with Alizarin red solution for 30 min at room temperature. Subsequently, cells were washed extensively with distilled water and images were captured using an inverted microscope.

### Lentivirus production and infection

The plasmids, a NOR1 overexpression (LV-NOR1) and knock-down (sh-NOR1) plasmid and the corresponding empty vectors (LV-con and sh-NC), were constructed by Hanbio Biotechnology Co. Ltd (Wuhan, China). Overexpressed plasmid and knock-down plasmid were transfected into HEK293E cells along with PMD2G and PSPAX2. After 48–72 h of transfection, lentivirus particles in the culture supernatant were collected and centrifuged. Human PDLSCs were infected with lentivirus particles for 12 h using polybrene. After 48 h, cells in each group were observed under light and fluorescence microscopes. The ratio of GFP-labeled cells to total cells was quantified by imaging six fields of view under 20x magnification using a fluorescence microscope. The efficiency of NOR1 overexpression and knock-down were determined by RT-qPCR and western blot analysis.

### RNA-seq

PDLSCs were seeded on 6 well plate with the density of 1 × 10^5^ cells/well. After sub-confluence, cells were ground under liquid nitrogen in a mortar and pestle, and the resulting powder dissolved in TRIzol™ Reagent (Invitrogen, Carlsbad, CA, USA), then extracted total RNA. The quality and purity of RNA were examined by a NanoDrop™ One/OneC spectrophotometer (Thermo Scientific, Waltham, MA, USA) and Life Invitrogen Qubit RNA BR (Broad-Range) Assay Kit. RNA integrity was analyzed using Agilent 4200 TapeStation system (Agilent, Santa Clara, CA, USA). 0.1-1 µg of total RNA per sample was prepared for library preparation. The NEBNext^®^ Poly(A) mRNA Magnetic Isolation Module and NEBNext^®^ Ultra™ II mRNA Library Prep Kit for Illumina^®^ were used for mRNA isolation and library construction following the manufacturer’s protocols. As for the quality control of library, Qubit dsDNA HS Assay Kit was used to measure the concentration of library, then Agilent 4200 was used to examine the distribution of segments in library. Finally, library molar concentration was determined using the KAPA Library Quant kit (illumina) universal qPCR Mix. High-throughput transcriptome sequencing was performed on an Illumina NovaSeq 6000 platform according to the manufacturer’ s instructions. Then, we assessed the quality of the raw sequencing data using FastQC and trimmed low-quality reads and adapter sequences using Trimmomatic v0.39. Clean reads were aligned to the human genome (GRCh38) using HISAT2 v2.2.1, and gene-level counts were obtained using featureCounts v2.0.1. Differential expression analysis was performed using DESeq2 v1.32.0. Genes with adjusted p-value < 0.05 was considered significantly differentially expressed. Functional and pathway enrichment analysis was conducted using clusterProfiler v4.2.0, with hypergeometric tests used to identify significantly enriched GO terms in GO enrichment analysis, and a significance threshold set at p-value < 0.05 for KEGG pathway enrichment analysis.

### ChIP-seq

We analyzed the result of ChIP-seq published in Nature Communication by Florian Haller et al. (dataset accession number: EGAS00001002795) [20] to find out the direct downstream targets regulating by NOR1.

### Dual luciferase activity assay

3000 bp upstream of TGFBR1 was inserted into the pGL3-basic plasmid. Plasmids overexpressing NOR1 and the corresponding empty vector were purchased from genechem Co. Ltd. (Shanghai, China). The promoter plasmid was co-transfected into HEK293E cells with NOR1 overexpressed plasmid and pRL-TK plasmid using lipofectamine 3000 (Invitrogen, USA). After 36–48 h of transfection, cells were lysed and measured using the Dual-Luciferase^®^ Assay System (Promega, USA) according to the manufacturer’s instructions. Renilla luciferase was used as an internal control.

### ChIP-qPCR

Chromatin immunoprecipitation was performed using EZ-ChIP™ chromatin immunoprecipitation kit (Millipore, Billerica, MA, USA) according to the manufacturer’s instructions. In brief, PDLSCs at a density of 1 × 10^6^ cells/dish were cross-linked by 1% formaldehyde. Then, the cells were collected and suspended in lysis buffer. The cross-linked DNA were sonicated into 200–1000 bp fragment. Protein G agarose was added into diluted DNA buffer and then centrifuged and followed by adding anti-NOR1 (Proteintech Group, Inc, Rosemont, USA) or IgG antibody (Proteintech Group, Inc, Rosemont, USA). The complex of protein and DNA were eluted and purified. Lastly, the purified DNA was analyzed by qPCR. The primers for qPCR were listed in Supplementary Table 1.

*Hematoxylin and eosin (H&E) staining and immunohistochemistry (IHC).*

Healthy third molars were collected from the department of stomatology, Peking University Shenzhen Hospital. The teeth were fixed with 4% paraformaldehyde for 24 h and were decalcified using 10% EDTA for 10 months, followed by dehydrating and emdedding in paraffin. All specimens were cut into 5 μm sections. And slices were dried, deparaffinized and rehydrated. Hematoxylin and eosin staining was used for histological and histomorphometric analysis. To detect the expression of NOR1 in periodontal ligament tissue, immunohistochemical staining was used according to the manufacturer’s instruction (Zhong Shan Biotech, Beijing, China). Briefly, the slides were treated with citrate for 24 h for antigen repair, and then incubated with 3% hydrogen peroxide to eliminate endogenous peroxidase. After that, slides were rinsed with PBS for three times, blocked with sheep serum for 1 h, incubated with primary antibody against NOR1 (1:200, Abcam, Cambridge, UK), overnight. Next day, the slices were washed with PBS three times and incubated with appropriate second antibody for 1 h at room temperature. Subsequently, the expression of NOR1 was detected using the DAB kit (Maixin Biotech. Co., Ltd, Fuzhou, China) according to the manufacturer’s instruction.

### Statistical analysis

All data in this study were analyzed using GraphPad Prism 6 Software. Statistical differences were assessed by unpaired Student’s t-test for two groups after Shapiro-Wilk test for the normal distribution of values. And one-way analysis of variance (ANOVA) was used for three groups, followed by Tukey’s *post hoc* test as appropriate. All data are presented as mean ± standard deviation (SD), as indicated in figure legends. A value of *P* < 0.05 was considered statistically significant.

## Results

### NOR1 is expressed in PDL tissue and upregulated during osteogenic differentiation of human PDLSCs

**Fig. 1:**
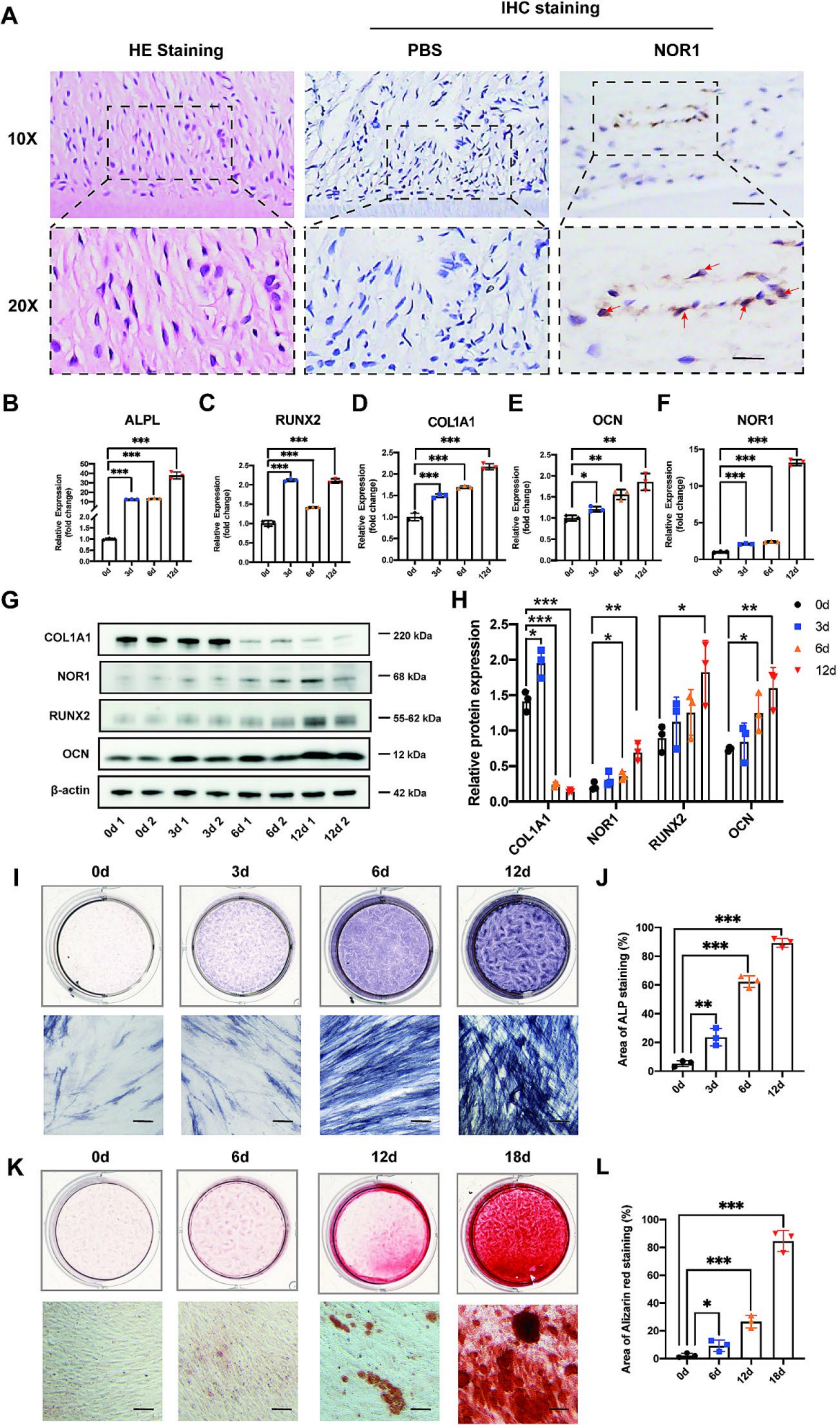
NOR1 is expressed in PDL tissue and upregulated during osteogenic differentiation of human PDLSCs. (**A**) Representative images of HE staining of PDL tissue sections from human teeth and IHC staining of NOR1 expression. Red arrows indicate NOR1 positive cells. Scale bar, 100 μm and 50 μm. (**B**-**F**) RT-qPCR analysis of NOR1 and osteogenic markers, ALPL, RUNX2, COL1A1, OCN expression in PDLSCs after osteogenic induction for 0, 3, 6, 12d. *n* = 3. *P < 0.05, **P < 0.01, ***P < 0.001, *versus* controls. One-way ANOVA, followed by Tukey multiple comparisons post test. (**G**) Two replicates of western-blot analysis of NOR1 and osteogenic genes, RUNX2, OCN, COL1A1 expression in PDLSCs during osteogenic differentiation. (**H**) Quantification of the relative protein expression. *n* = 3. *P < 0.05, **P < 0.01, ***P < 0.001, *versus* controls. One-way ANOVA, followed by Tukey multiple comparisons post test. (**I**) ALP staining of PDLSCs with osteogenic induction for 0, 3, 6, 12d. Scale bar, 200 μm. (**J**) Quantification of ALP positive area. *n* = 3. *P < 0.05, **P < 0.01, ***P < 0.001, *versus* controls. One-way ANOVA, followed by Tukey multiple comparisons post test. (**K**) Alizarin red staining of PDLSCs with osteogenic induction for 0, 6, 12, 18d. Scale bar, 200 μm. (**L**) Quantification of Alizarin red staining. *n* = 3. *P < 0.05, **P < 0.01, ***P < 0.001, *versus* controls. One-way ANOVA, followed by Tukey multiple comparisons post test

As shown in Fig. 1A, HE staining showed the nornal morphology of PDL tissue. IHC staining revealed that NOR1 was expressed extensively in PDL tissue and located in periodontal cell nucleus. Subsequently, we isolated PDLSCs from PDL tissue. The result of immunofluoresence staining showed that cultured PDLSCs were positive expression of mesenchymal cell marker, vimentin, while negative expression of epithelial cell marker, pan cytokeratin (PCK) (Supplementary Fig. 1A). Next, we used flow cytometry to detect surface markers of PDLSCs. As can be seen in Supplementary Fig. 1B-G, PDLSCs express markers of MSCs, including CD44 (99.5%), CD105 (97.8%) and are negative for HLA-DR (0.17%), CD45 (0.02%), CD34 (0.34%). Further, cells were cultured with osteogenic and adipogenic medium. After osteogenic induction for 21d, PDLSCs exhibited calcific nodules formation (Supplementary Fig. 1H, 1I). After adipogenic induction for 18d, cells showed a large amount of lipid accumulation (Supplementary Fig. 1J, 1 K). Taken together, these results indicated that cultured PDLSCs are mesenchymal origin with the ability of multi-lineage differentiation.

To detect the expression of NOR1 during osteogenic differentiation of PDLSCs in vitro, we collected RNA and protein sample of PDLSCs induced for 0, 3, 6, 12d. The results of RT-qPCR showed that the expression of osteogenic markers, alkaline phosphatase (ALPL), RUNX family transcription factor 2 (RUNX2), type I (a) collagen (COL1A1) and osteocalcin (OCN), were increased after mineralization induction (Fig. 1B-E). Interestingly, NOR1 expression was also gradually increased during the osteogenic differentiation of PDLSCs (Fig. 1F). Additionally, western-blot analysis and quantitative results of RUNX2, OCN, NOR1 expression were consistent with that of RT-qPCR assays (Fig. 1G and H). However, the expression of COL1A1 protein was upregulated at day 3 of osteogenic induction but was downregulated after osteogenic induction for 6d and 12d (Fig. 1G and H). Additionally, ALP staining showed increased ALP expression levels in PDLSCs following the induction of mineralization (Fig. 1I and J). Furthermore, calcium deposits and mineralized nodule formation in PDLSCs were steadily increased after osteogenic induction for 6d, 12d and 18d (Fig. 1K and L). The above results suggested that NOR1 may be of importance in regulating the process of the osteogenic differentiation of PDLSCs.

### Knockdown of NOR1 reduces osteoblast differentiation while NOR1 overexpression increases the osteogenic potential of PDLSCs

**Fig. 2:**
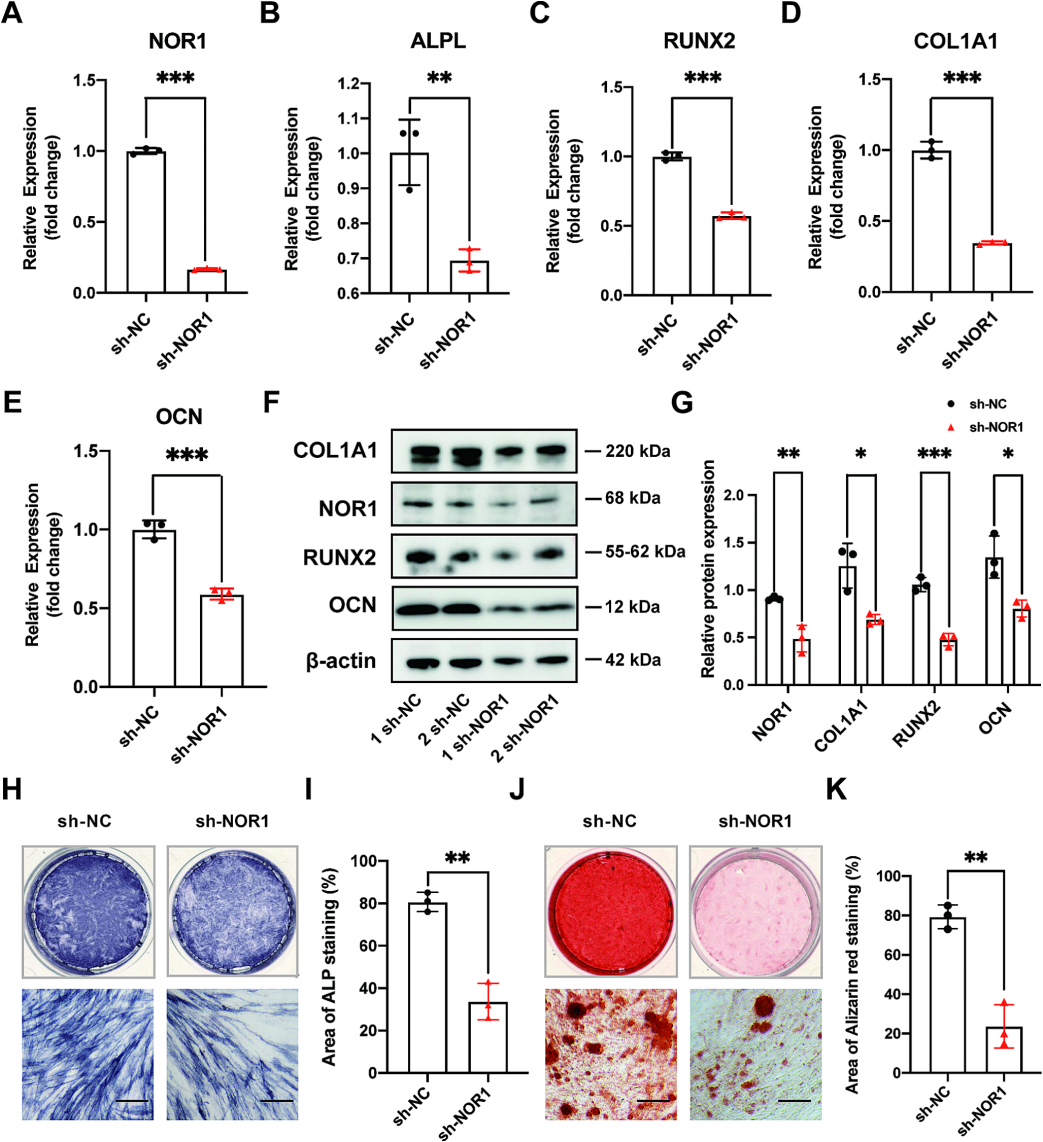
Knockdown of NOR1 inhibits the osteogenic differentiation potential of PDLSCs. (**A**) The efficiency of NOR1 knock-down by the shRNA was confirmed by RT-qPCR (sh-NOR1, cells transfected with lentiviral vector containing NOR1 knock-down sequence; sh-NC, control cells transfected with a unordered sequence). *n* = 3. *P < 0.05, **P < 0.01, ***P < 0.001, *versus* controls. Student’s t test. Data are presented as mean ± standard deviation (SD). (**B**-**E**) RT-qPCR analysis of osteogenic markers, ALPL, RUNX2, COL1A1 and OCN expression after NOR1 knocking down in PDLSCs. *n* = 3. *P < 0.05, **P < 0.01, ***P < 0.001, *versus* controls. Student’s t test. Data are presented as mean ± standard deviation (SD). (**F**) Two replicates of western-blot analysis of NOR1, RUNX2, OCN, COL1A1 expression in PDLSCs with NOR1 knocking down. (**G**) Quantification of the relative protein expression. *n* = 3. *P < 0.05, **P < 0.01, ***P < 0.001, *versus* controls. Student’s t test. Data are presented as mean ± standard deviation (SD). (**H**) ALP staining showed that NOR1 knock-down decreased the ALP activity of PDLSCs. Scale bar, 200 μm. (**I**) Quantification of ALP staining. *n* = 3. *P < 0.05, **P < 0.01, ***P < 0.001, *versus* controls. Student’s t test. Data are presented as mean ± standard deviation (SD). (**J**) Alizarin red staining implied that NOR1 knock-down inhibited the calcium nodules formation in PDLSCs. Scale bar, 200 μm. (**K**) Quantification of Alizarin red staining. *n* = 3. *P < 0.05, **P < 0.01, ***P < 0.001, *versus* controls. Student’s t test. Data are presented as mean ± standard deviation (SD)

To explore the function of NOR1 in the process of osteogenic differentiation of PDLSCs, we designed and constructed NOR1 shRNA to knock down its endogenous expression. The results of RT-qPCR showed that the expression of NOR1 and osteogenic genes, ALPL, RUNX2, COL1A1, OCN, were all downregulated in PDLSCs after NOR1 knocking down (Fig. 2A-E). The protein level of RUNX2, OCN and COL1A1 were also decreased upon the low expression of NOR1 in PDLSCs (Fig. 2F and G). After osteogenic induction for 7d, the result of ALP staining showed that NOR1 knocking down decreased the osteogenic potential of PDLSCs (Fig. 2H and I). After 21d, the result of Alizarin red staining indicated that knockdown of NOR1 in PDLSCs resulted in the decrease of calcific nodules formation (Fig. 2J and K).

**Fig. 3:**
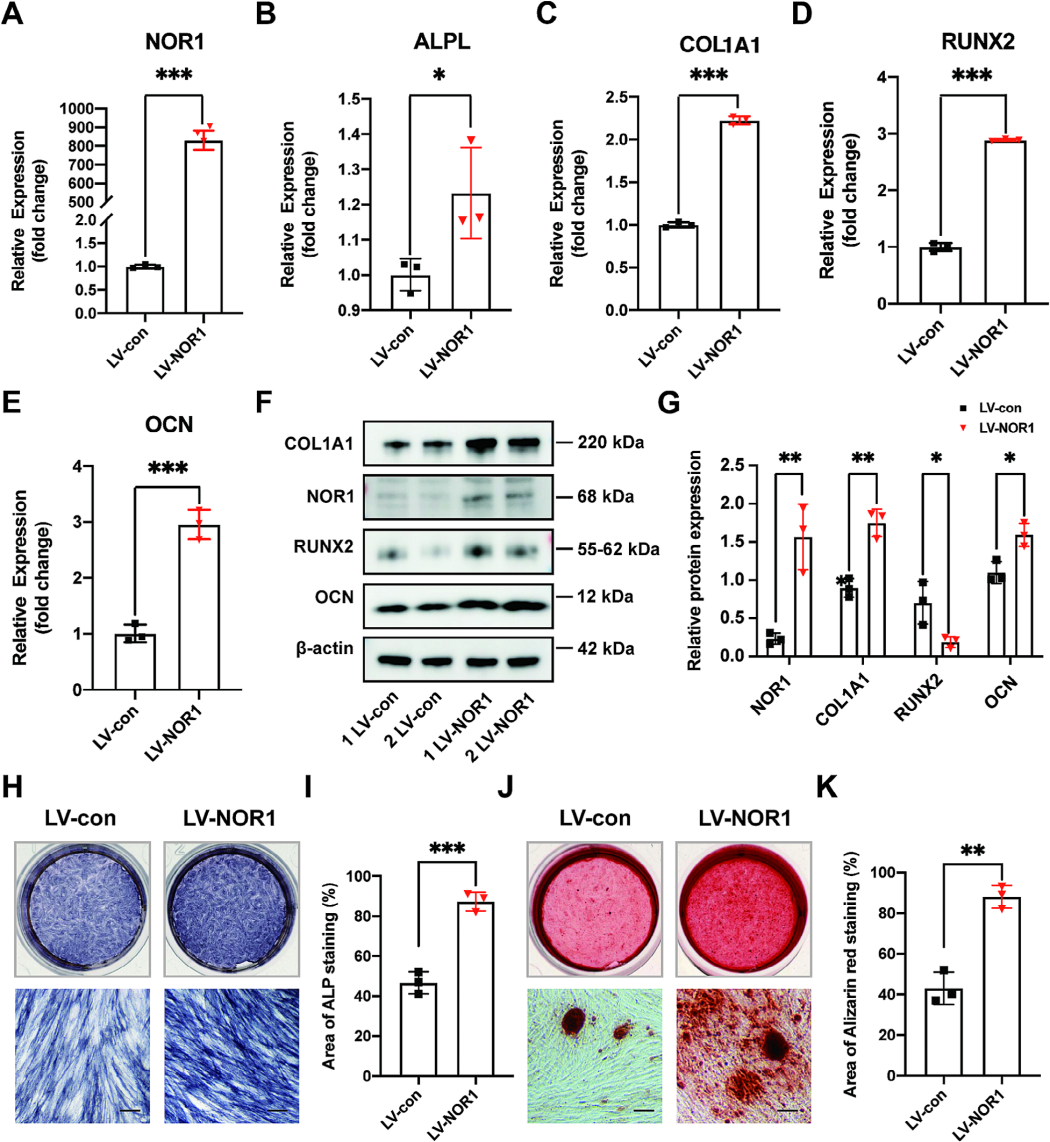
NOR1 overexpression promotes the osteoblast differentiation of PDLSCs. (**A**) The efficiency of NOR1 overexpression by the lentiviral vector transfection was confirmed by RT-qPCR (LV-NOR1, cells transfected with lentiviral vector expressing NOR1; LV-Con, control cells transfected with empty vector). *n* = 3. *P < 0.05, **P < 0.01, ***P < 0.001, *versus* controls. Student’s t test. Data are presented as mean ± standard deviation (SD). (**B**-**E**) Relative mRNA expression levels of the osteogenic genes, ALPL, COL1A1, RUNX2 and OCN in PDLSCs overexpressing NOR1 compared to those in controls transfected with empty vector. *n* = 3. *P < 0.05, **P < 0.01, ***P < 0.001, *versus* controls. Student’s t test. Data are presented as mean ± standard deviation (SD). (**F**) Two replicates of western-blot analysis of NOR1, RUNX2, OCN expression in PDLSCs after NOR1 overexpression. (**G**) Quantification of the relative protein expression. *n* = 3. *P < 0.05, **P < 0.01, ***P < 0.001, *versus* controls. Student’s t test. Data are presented as mean ± standard deviation (SD). (**H**) ALP staining showed NOR1 overexpression enhanced the ALP activity of PDLSCs. Scale bar, 200 μm. (**I**) Quantification of ALP staining. *n* = 3. *P < 0.05, **P < 0.01, ***P < 0.001, *versus* controls. Student’s t test. Data are presented as mean ± standard deviation (SD). (**J**) Alizarin red staining revealed more calcium nodules formation in PDLSCs transfected with NOR1 overexpressed vector compared to those transfected with empty vector. Scale bar, 200 μm. (**K**) Quantification of Alizarin red staining. *n* = 3. *P < 0.05, **P < 0.01, ***P < 0.001, *versus* controls. Student’s t test. Data are presented as mean ± standard deviation (SD)

Additionally, we constructed NOR1 overexpression plasmid, packaged in lentiviral particle and transfected in PDLSCs. The results of RT-qPCR indicated osteogenesis-related genes expression were increased after NOR1 overexpression (Fig. 3A-E), which is contrary to the results of NOR1 knocking down. The western blot analysis and quantitative results of RUNX2, OCN and COL1A1 expression were consistent with that of the RT-qPCR assays (Fig. 3F and G). Furthermore, we can see enhanced ALP activity (Fig. 3H and I) and more calcific nodules formation (Fig. 3J and K) in PDLSCs with NOR1 overexpression.

Taken together, we concluded that NOR1 could regulate the process of osteogenic differentiation of PDLSCs. When the expression of NOR1 is low in PDLSCs, osteogenic differentiation of PDLSCs is weakened while the osteogenic potential is enhanced after NOR1 overexpression.

### NOR1 knockdown inhibits the activation of TGF-β/SMAD signaling pathway during osteoblast differentiation of PDLSCs

**Fig. 4:**
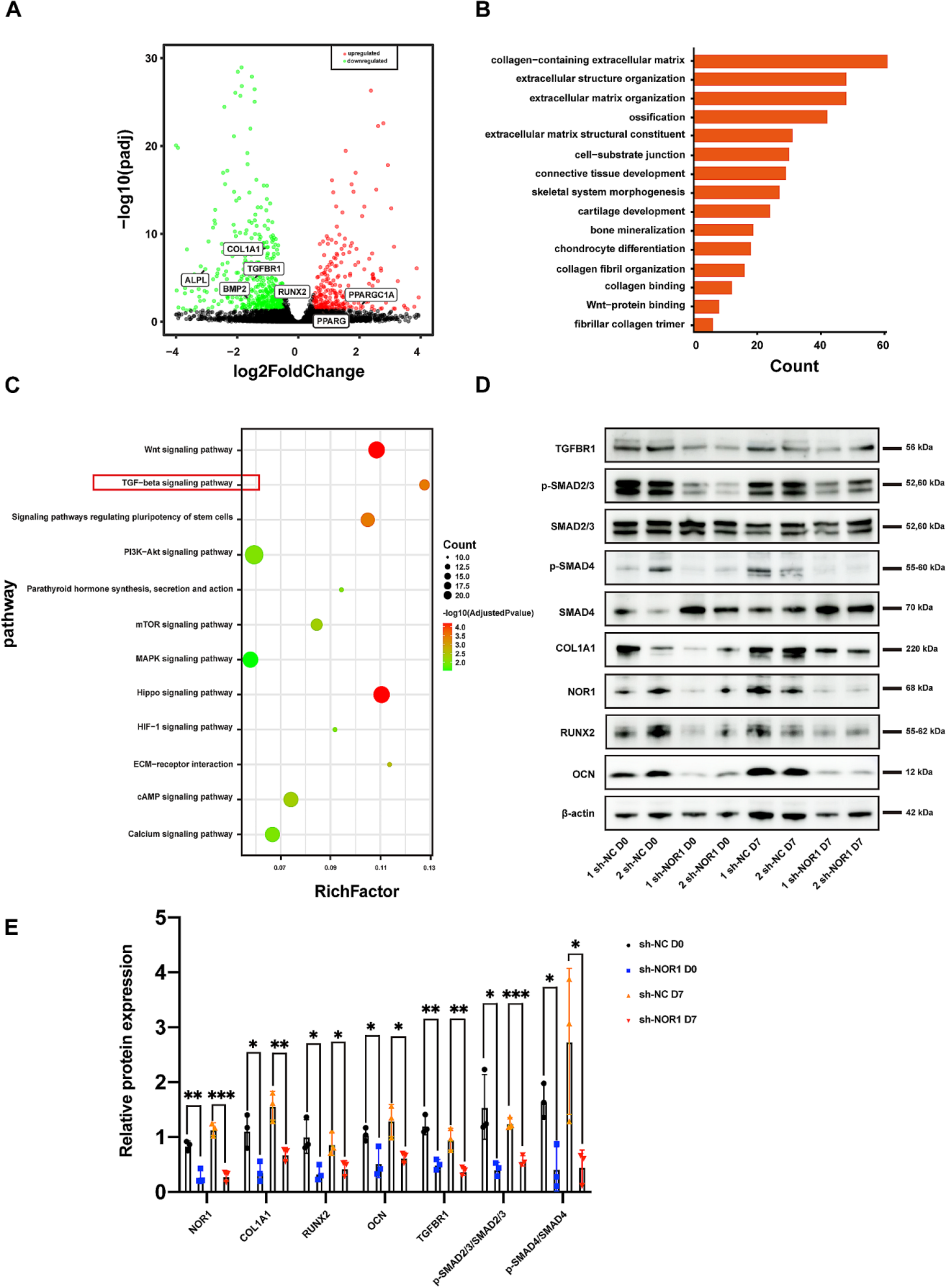
Genomewide profile of NOR1-regulatory genes during osteoblast differentiation of PDLSCs.(**A**) The scatter plot showed the up-regulated and down-regulated genes by sh-NOR1 transfection in PDLSCs. Red dots represent significantly upregulated genes in PDLSCs with NOR1 knock-down compared with the control cells. Green dots represent significantly downregulated genes in PDLSCs with NOR1 knock-down compared with the control cells. Black dots represent equally-expressed genes. (**B**) GO enrichment analysis of down-regulated genes by sh-NOR1 in PDLSCs. (**C**) Top 12 enriched pathways are shown in the scatter plot. (**D**) Two replicates of western blot analysis of TGFBR1, p-SMAD 2/3, SMAD 2/3, p-SMAD4, SMAD4, RUNX2, OCN, COL1A1 and NOR1 expression in PDLSCs by NOR1 knocking down with or without osteogenic induction. (**E**) Quantification of the relative protein expression. *n* = 3. *P < 0.05, **P < 0.01, ***P < 0.001, *versus* controls. Student’s t test. Data are presented as mean ± standard deviation (SD)

To further reveal the NOR1-related regulatory mechanisms during osteoblast differentiation of PDLSCs, we conducted RNA-seq in PDLSCs transfected with sh-NOR1 or scramble shRNA. According to our RNA-seq result, the expression of NOR1 was obviously downregulated in PDLSCs transfected with sh-NOR1 for 72 h (Fig. 4A). Totally, 897 genes were identified differentially expressed between knock-down group and control group. Among them, 341 genes were significantly upregulated while 556 genes were downregulated followed by sh-NOR1 transfection. The significantly downregulated genes including RUNX2, COL1A1, ALPL, BMP2, which are all osteogenic markers (Fig. 4A). Conversely, the expression of adipogenic markers PPARG and PPARPGA were upregulated (Fig. 4A).

Additionally, GO enrichment analysis showed that the downregulated genes were notably enriched in mineralization related GO term, including ossification (GO:0001503, q value = 3.46930493033829E-10) and bone mineralization (GO:0030282, q value = 2.94717652518901E-07), which indicated that NOR1 exerted positive effect on osteogenic differentiation and mineralization (Fig. 4B). Furthermore, KEGG analysis found that the downregulated genes by sh-NOR1 transfection were enriched in TGF-β signaling pathway (Fig. 4C). TGF-β signaling is indispensable for various physiological and pathological process, including osteoblast differentiation and bone formation [21, 22]. Specifically, upon ligand binding, TGFBR1 activates SMAD2 and SMAD3 through phosphorylation. Then, phosphorylated SMAD2 and SMAD3 cooperate with SMAD4 and form trimeric complexes, which are translocated into nucleus and regulate the transcription of downstream targets [23, 24]. At present, we found that the expression of main components of TGF-β signaling pathway, TGFBR1, phosphlyrated SMAD2/3 (p-SMAD2/3) and phosphlyrated SMAD4 (p-SMAD4), were significantly downregulated afer NOR1 knocking down in PDLSCs with or without osteogenic induction (Fig. 4D and E). However, sh-NOR1 transfection in PDLSCs had no effect on the expression of the inhibitory SMAD, SMAD7 (Supplementary Fig. 2A, 2B). Therefore, NOR1 knockdown had a negative effect on TGF-β/SMAD signaling pathway during osteoblast differentiation of PDLSCs.

### NOR1 modulates osteogenesis of PDLSCs by targeting TGFBR1 directly

**Fig. 5:**
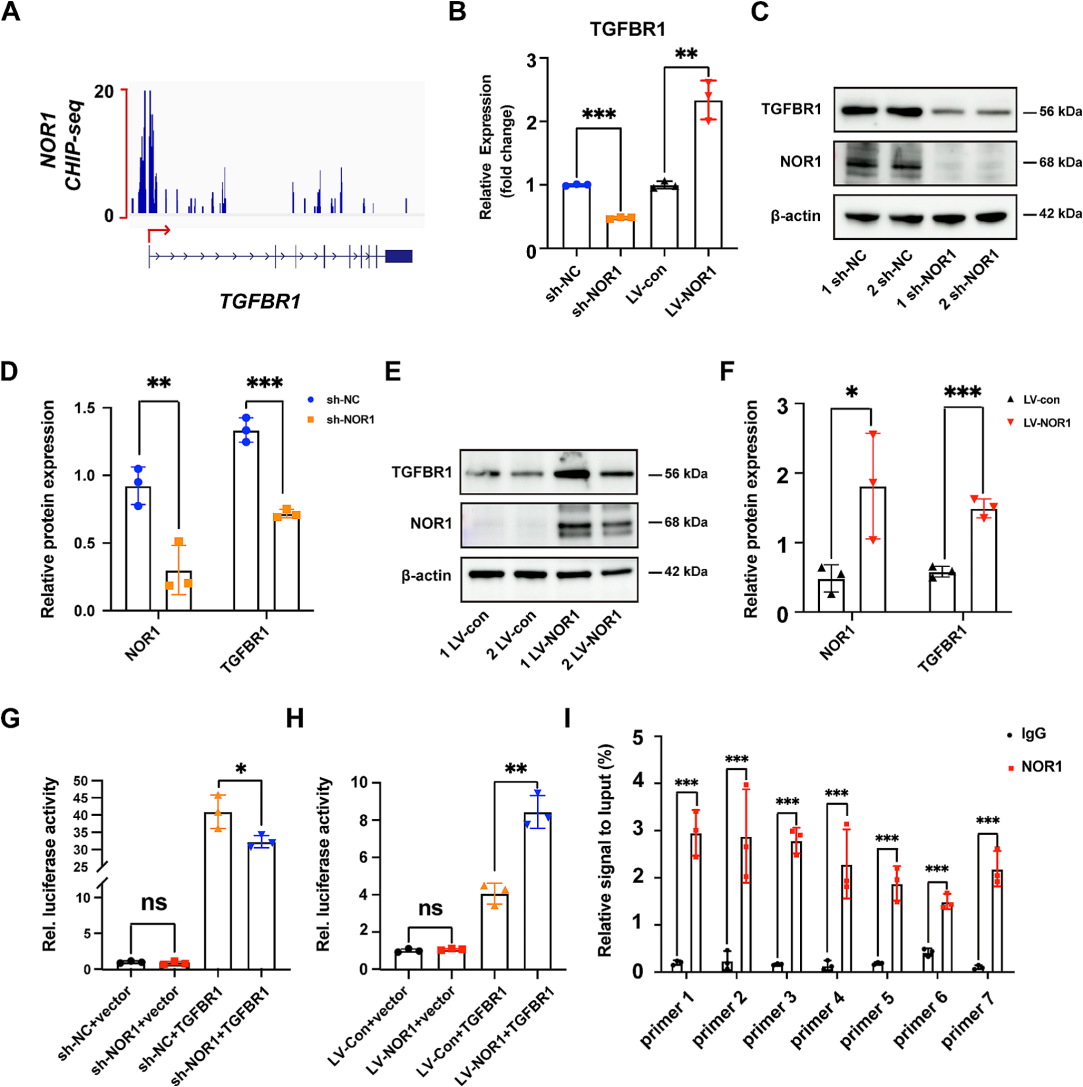
NOR1 directly activates TGFBR1. (**A**) Analysis of ChIP-seq revealed that the peaks of NOR1 enriched in the promoter of TGFBR1. (**B**) RT-qPCR analysis of TGFBR1 expression in PDLSCs with NOR1 knock-down or overexpression. *n* = 3. *P < 0.05, **P < 0.01, ***P < 0.001, *versus* controls. Student’s t test. Data are presented as mean ± standard deviation (SD). (**C**) Two replicates of western blot analysis of TGFBR1 expression in PDLSCs with NOR1 knock-down. (**D**) Quantification of the relative protein expression. *n* = 3. *P < 0.05, **P < 0.01, ***P < 0.001, *versus* controls. Student’s t test. Data are presented as mean ± standard deviation (SD). (**E**) Two replicates of western blot analysis of TGFBR1 expression in PDLSCs with NOR1 overexpression. (**F**) Quantification of the relative protein expression. *n* = 3. *P < 0.05, **P < 0.01, ***P < 0.001, *versus* controls. Student’s t test. Data are presented as mean ± standard deviation (SD). The dual-luciferase reporter assay demonstrated that sh-NOR1 significantly reduced the promoter activity of TGFBR1 (**G**) while NOR1 overexpression significantly increased the transcriptional activity of TGFBR1 promoter (**H**). *n* = 3. *P < 0.05, **P < 0.01, ***P < 0.001, *versus* controls. Student’s t test. Data are presented as mean ± standard deviation (SD). (**I**) ChIP-qPCR analysis of NOR1 binding to the promoter of TGFBR1. *n* = 3. *P < 0.05, **P < 0.01, ***P < 0.001, *versus* controls. Student’s t test. Data are presented as mean ± standard deviation (SD)

According to the result of Florian Haller et al’s, there is a significant enrichment of NOR1 peaks observed in the promoter region of TGFBR1 [20] (Fig. 5A). Our results of RT-qPCR indicated the expression of TGFBR1 is decreased upon NOR1 knocking down and is increased when NOR1 is overexpressed in PDLSCs (Fig. 5B). The results of western blot analysis and quantitative results are consistent with that of RT-qPCR assays (Fig. 5C-F). Importantly, dual luciferase reporter assay showed that sh-NOR1 reduced the promoter activity of TGFBR1 compared with transfection with mutant sequence (Fig. 5G) while NOR1 overexpression significantly increased the transcriptional activity of TGFBR1 promoter (Fig. 5H). Subsequently, ChIP-qPCR was used to examined the direct binding of NOR1 to the promoter region of TGFBR1 using the specific primer sets listed in Supplementary material. As shown in Fig. 5I, primers 1, 2, 3, 4, 5, 6 and 7 successfully amplified the corresponding PCR products from DNAs pulled down by NOR1 antibody. These results indicated that the transcriptional activity of TGFBR1 was modulated by NOR1

**Fig. 6:**
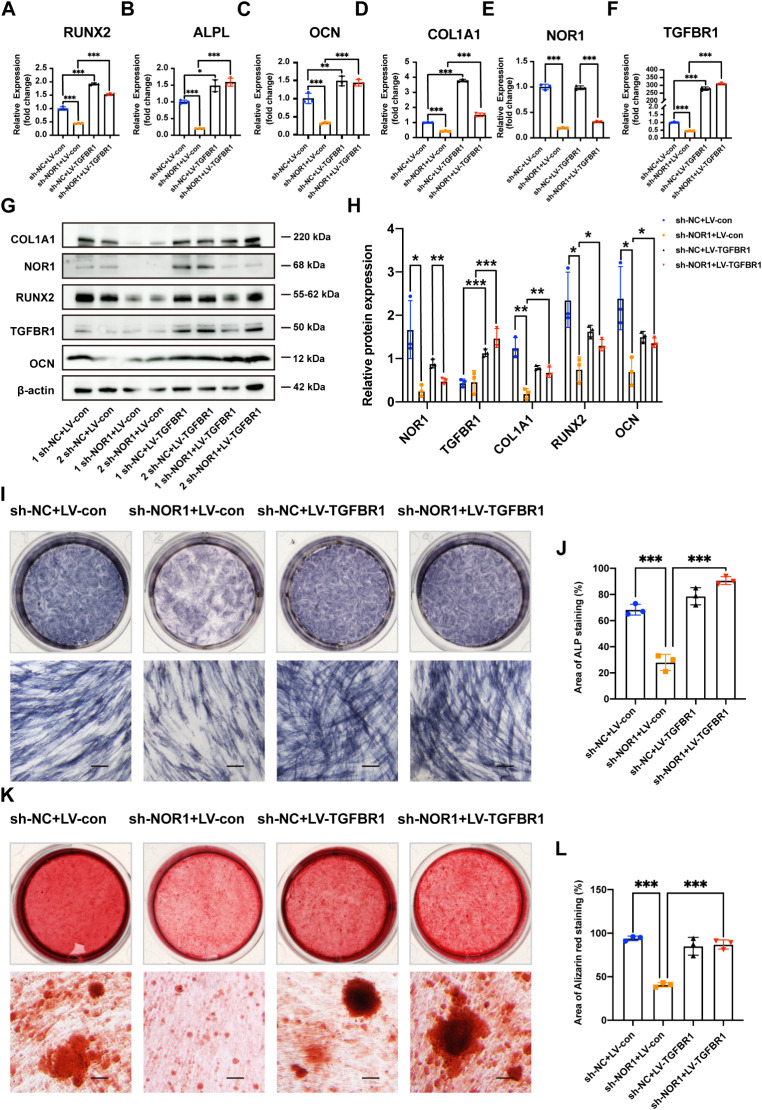
NOR1 modulates osteogenesis of PDLSCs through targeting TGFBR1 directly. (**A**-**D**) The results of RT-qPCR indicated that the decreased expression of osteogenic genes, RUNX2, ALPL, OCN, COL1A1 caused by NOR1 knockdown (**E**) were all rescued by TGFBR1 overexpression (**F**). *n* = 3. *P < 0.05, **P < 0.01, ***P < 0.001, *versus* controls. One-way ANOVA, followed by Tukey multiple comparisons post test. (**G**) Two replicates of western blot analysis of COL1A1, RUNX2, OCN expression in PDLSCs with NOR1 knock-down and TGFBR1 overexpression. (**H**) Quantification of the relative protein expression. *n* = 3. *P < 0.05, **P < 0.01, ***P < 0.001, *versus* controls. One-way ANOVA, followed by Tukey multiple comparisons post test. (**I**) ALP staining showed reversed osteogenic potential after TGFBR1 overexpression. Scale bar, 200 μm. (**J**) Quantification of ALP staining. *n* = 3. *P < 0.05, **P < 0.01, ***P < 0.001, *versus* controls. One-way ANOVA, followed by Tukey multiple comparisons post test. (**K**) The Alizarin red staining verified rescued mineralization ability due to TGFBR1 overexpression in PDLSCs with low NOR1 expression. Scale bar, 200 μm. (**L**) Quantification of Alizarin red staining. *n* = 3. *P < 0.05, **P < 0.01, ***P < 0.001, *versus* controls. One-way ANOVA, followed by Tukey multiple comparisons post test

Next, to explore whether NOR1 modulates osteoblast differentiation of PDLSCs through targeting TGFBR1 directly. We contructed TGFBR1 overexpression plasmid and transfected into PDLSCs with NOR1 knockdown. The results of RT-qPCR indicated that the decreased expression of osteogenic genes, RUNX2, ALPL, OCN and COL1A1(Fig. 6A-D) caused by NOR1 konckdown (Fig. 6E) were all rescued by TGFBR1 overexpression (Fig. 6F). The results of western blot analysis of RUNX2, OCN and COL1A1 expression were consistent with that of RT-qPCR assay (Fig. 6G and H). The ALP staining also showed reversed osteogenic capacity after TGFBR1 overexpression (Fig. 6I and J). The Alizarin red staining further verified rescued mineralization ability due to TGFBR1 transfection in PDLSCs with low NOR1 expression (Fig. 6K and L).

**Fig. 7:**
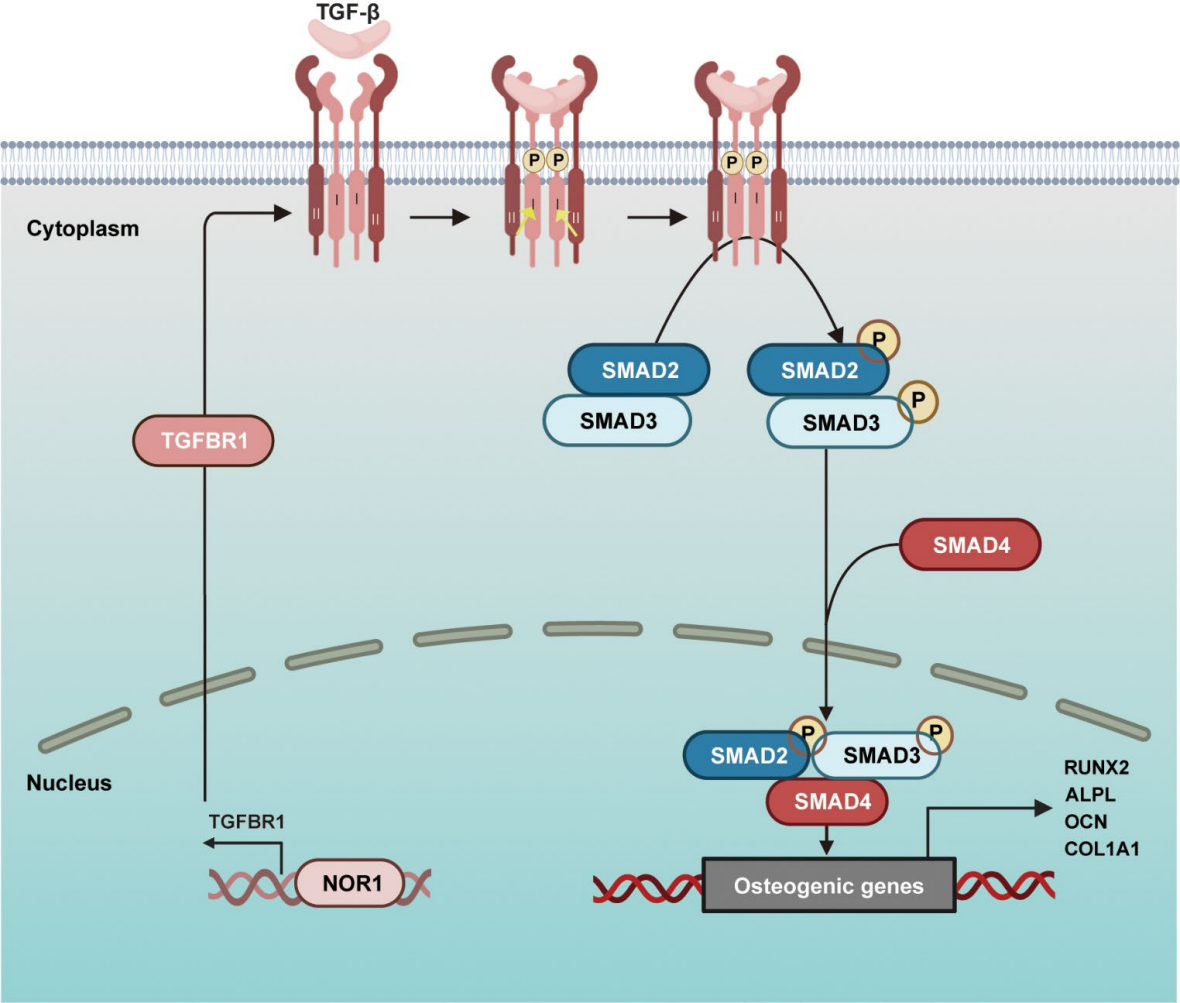
Schematic illustration of NOR1 regulating osteoblast differentiation of PDLSCs. NOR1 binds to the promoter region of TGFBR1 directly or regulates the TGF-β/SMAD signaling pathway to promote the osteoblast differentiation of PDLSCs

In summary, NOR1 modulates the osteoblast differentiation of PDLSCs through targeting TGFBR1 directly and may activate the TGF-β/SMAD signaling pathway (Fig. 7).

## Discussion

Transcription factors could regulate the expression of a large number of downstream genes and hence are considered as important regulator of osteogenesis. For PDLSCs, previous studies have revealed a multitude of transcription factors are involved in the osteoblast lineage commitment [25–27]. NOR1, a member of the NR4A subfamily of nuclear receptors, binds to the DNA sequence known as the NGFI-B (AAAGGTCA) response element to regulate cell proliferation [28], differentiation [29] and metabolism. However, there is still little knowledge about the biological role of NOR1 in osteoblast differentiation processes. At the present study, we for the first time found that NOR1 was expressed in PDL tissue and upregulated during osteogenic differentiation of PDLSCs. By using a strategy that combines gain- and loss-of function approaches, we found NOR1 is a positive regulator of osteogenic differentiation of PDLSCs. According to the bioinformatic analysis, there are potential NGFI-B response elements in the promoter region of several osteogenic markers, including ALPL, BSP, OCN, OPN and COL1A1. Except that, Nurr1, another member of NR4A subfamily of nuclear receptors which harbors the same DNA-binding domain and the ligand-binding domain with NOR1, induced OCN and OPN expression in osteoblasts via direct binding to and transactivation of their promoters [30, 31]. In PDLSCs, NOR1 may also regulate the expression of these osteogenic genes by direct binding to their promoter regions, but this need further exploration.

For MSCs, the osteogenic and adipogenic differentiation are considered to be a mutually exclusive process due to adipocytes and osteoblasts share a common progenitor source. Previously, a research demonstrated that knockdown of ASCC1 in hMSCs resulted in inhibited osteoblast differentiation while stimulated adipogenesis, which resulted in reduced mineralization and increased lipid droplets formation [32]. According to our results, NOR1 could promote osteogenesis of PDLSCs, but whether it promotes or inhibits the adipogenic differentiation is unclear. Interestingly, our RNA-seq results showed the adipogenic markers including PPARG and PPARPGA were upregulated after transfection with sh-NOR1 in PDLSCs. Based on this finding, we speculated that NOR1 inhibits the adipogenic differentiation and shift the balance of osteoblast differentiation and adipogenic differentiation of PDLSCs into osteogensis. In fact, other researchers have demonstrated NOR1 modulates the adipogenic process [33–35]. For example, it is reported that the adipogenic potential of pre-adipocyte 3T3-L1 or 3T3-F442A were inhibited obviously after NOR1 overexpression using retrovirus technology [13]. In the future study, we will focus on the role and mechanism of NOR1 modulating the adipogenesis of PDLSCs deeply and validating our hypothesis further.

TGF-β signaling is indispensable for bone formation and skeleton development. It has been indicated that TGF-β signaling-dificient mice including Tgfb1-null mice, MSC-specific and osteoprogenitor-specific Tgfbr2 CKO mice exhibited significant bone loss with reduced osteoblast number [36–38]. The enrichment analysis results of KEGG pathways in our study showed that TGF-β signaling pathway is enriched in sh-NOR1 transfected PDLSCs. Further, we proved the activation of SMAD2/3 and SMAD4 were both inhibited by NOR1 kocking down. These results verified TGF-β signaling pathway was involved in the process of NOR1 regulating the osteogenic differentiation of PDLSCs. However, we have not administrated the activator or inhibitor of TGF-β signaling pathway after NOR1 knocking down or overexpression, so we can not confirm the direct interaction between NOR1 and TGF-β signaling pathway. Additionally, the controversial viewpoints of TGF-β signaling in different stages of osteogenic differentiation exist among some researches. Several studies indicated that TGF-β could promote early differentiation and matrix production of osteoblast progenitor cells, but inhibit the later differentiation and mineralization [39, 40]. In these studies, TGF-β is deemed as the early inducer of the differentiation of osteoblasts from MSCs. However, a study on porcine SMSCs revealed that TGF-β signaling pathway was mainly enriched in the middle and later periods of osteogenic differentiation [41]. At the present study, we just observed a short time but not detected a longer period of osteogenic differentiation after sh-NOR1 transfection in PDLSCs. It is meaningful to further explore the role of TGF-β signaling pathway in osteoblast differentiation of PDLSCs in vitro and in vivo.

TGFBR1 is a serine and threonine kinase receptor, which transforms TGF-β signaling. Previously, a result of ChIP-seq showed a significant enrichment of NOR1 peaks in the promoter region of TGFBR1 [20]. In fact, TGFBR1 is necessary for osteogenic differentiation. A previous study reported that loss of TGFBR1 in mice primary neonatal calvarial cells leads to inhibition of osteogenic differentiation [42]. Additionally, inhibition of TGFBR1 down-regulates osteoblast differentiation and mineralization of human MSCs [43]. At present, we have confirmed TGFBR1 is a direct downstream target of NOR1 and TGFBR1 overexpression could rescue the damaged osteogenic potential caused by NOR1 knocking down in PDLSCs. According to the literatures, upon phosphorylated, TGFBR1 then phosphorylates either downstream SMAD2/3 or kinases that activate other pathways, including MAPKs, ERK, P38, JNK and PI3K/PKB [44]. Therefore, we put forward two hypotheses for the function of TGFBR1 in the process of NOR1 modulating osteoblast differentiation of PDLSCs. One is that NOR1 induces the activation of SMAD 2/3 through TGFBR1 and then leads to osteoblast differentiation of PDLSCs. The other is that NOR1 modulates the amount and activity of TGFBR1, then activates downstream noncanonical signalings including MAPKs or PI3K pathways to coordinate the osteoblast commitment.

Although we have verified that NOR1 is a novel transcription factor of osteogenesis of PDLSCs, we also need acknowledge that there are some limitations in the present study. Firstly, a study on porcine SMSCs indicated that the genetic background of donor MSCs exhibited differences in osteogenesis [41], but in our study, we mixed the PDL tissue from different donors to isolate human PDLSCs, which may veil the individual differences. Secondly, our RNA-seq results revealed that the downregulated genes in PDLSCs with NOR1 knocking down are also enriched in Wnt signaling pathway and Hippo signaling pathway, which are also reported to be involved in osteogenesis widely [45, 46]. TGF-β/SMAD signaling is deemed to crosstalk with these signaling pathways to regulate various biological responses [47]. However, we did not elucidate the crosstalk among these signaling pathways in the process of NOR1 regulating osteoblast differentiation of PDLSCs. Lastly, but definitely not least, most conclusions in our study from the in vitro cell experiments, the animal model should be applied in our future study.

## Conclusion

In conclusion, we have successfully identified NOR1 as a positive regulator of osteogenesis of PDLSCs. Mechanistically, sh-NOR1 downregulates TGF-β/SMAD signaling pathway. In addition, we found NOR1 could bind to the promoter of TGFBR1 directly, modulating its activity and then coordinate the osteogenic process. Our findings provide new ideas for stem cell-based periodontal bone tissue engineering. Also, our results offer a new therapeutic target for bone loss-related diseases, such as osteoporosis and periodontitis.

### Electronic supplementary material

Below is the link to the electronic supplementary material.

Supplementary Material 1

Supplementary Material 2

Supplementary Material 3

Supplementary Material 4

Supplementary Material 5

## Data Availability

The data supporting the findings of the current study are available from the corresponding author upon reasonable request.

## References

[1] Cho MI, Garant PR (2000) Development and general structure of the periodontium. Periodontol 2000 24:9–27 11276876 10.1034/j.1600-0757.2000.2240102.x

[2] Payne KF, Balasundaram I, Deb S, Di Silvio L, Fan KF (2014) Tissue engineering technology and its possible applications in oral and maxillofacial surgery. Br J Oral Maxillofac Surg 52(1):7–15 23601833 10.1016/j.bjoms.2013.03.005

[3] Seo BM, Miura M, Gronthos S, Bartold PM, Batouli S, Brahim J et al (2004) Investigation of multipotent postnatal stem cells from human periodontal ligament. Lancet 364(9429):149–155 15246727 10.1016/S0140-6736(04)16627-0

[4] Iwata T, Yamato M, Washio K, Yoshida T, Tsumanuma Y, Yamada A et al (2018) Periodontal regeneration with autologous periodontal ligament-derived cell sheets - a safety and efficacy study in ten patients. Regen Ther 9:38–44 30525074 10.1016/j.reth.2018.07.002 PMC6222282

[5] Kronenberg HM (2003) Developmental regulation of the growth plate. Nature 423(6937):332–336 12748651 10.1038/nature01657

[6] Satokata I, Ma L, Ohshima H, Bei M, Woo I, Nishizawa K et al (2000) Msx2 deficiency in mice causes pleiotropic defects in bone growth and ectodermal organ formation. Nat Genet 24(4):391–395 10742104 10.1038/74231

[7] Tang T, Zhu Z, He Z, Wang F, Chen H, Liu S et al (2023) DLX5 regulates the osteogenic differentiation of spinal ligaments cells derived from ossification of the posterior longitudinal ligament patients via NOTCH signaling. JOR Spine 6(2):e1247 37361333 10.1002/jsp2.1247 PMC10285757

[8] Yang X, Matsuda K, Bialek P, Jacquot S, Masuoka HC, Schinke T et al (2004) ATF4 is a substrate of RSK2 and an essential regulator of osteoblast biology; implication for coffin-Lowry Syndrome. Cell 117(3):387–398 15109498 10.1016/S0092-8674(04)00344-7

[9] Zhao Y, Bruemmer D (2010) NR4A orphan nuclear receptors: transcriptional regulators of gene expression in metabolism and vascular biology. Arterioscler Thromb Vasc Biol 30(8):1535–1541 20631354 10.1161/ATVBAHA.109.191163 PMC2907171

[10] De Paoli F, Eeckhoute J, Copin C, Vanhoutte J, Duhem C, Derudas B et al (2015) The neuron-derived orphan receptor 1 (NOR1) is induced upon human alternative macrophage polarization and stimulates the expression of markers of the M2 phenotype. Atherosclerosis 241(1):18–26 25941992 10.1016/j.atherosclerosis.2015.04.798

[11] Calvayrac O, Rodríguez-Calvo R, Martí-Pamies I, Alonso J, Ferrán B, Aguiló S et al (2015) NOR-1 modulates the inflammatory response of vascular smooth muscle cells by preventing NFκB activation. J Mol Cell Cardiol 80:34–44 25536180 10.1016/j.yjmcc.2014.12.015

[12] Qing H, Jones KL, Heywood EB, Lu H, Daugherty A, Bruemmer D (2017) Deletion of the NR4A nuclear receptor NOR1 in hematopoietic stem cells reduces inflammation but not abdominal aortic aneurysm formation. BMC Cardiovasc Disord 17(1):271 29047330 10.1186/s12872-017-0701-4 PMC5648424

[13] Chao LC, Bensinger SJ, Villanueva CJ, Wroblewski K, Tontonoz P (2008) Inhibition of adipocyte differentiation by Nur77, Nurr1, and Nor1. Mol Endocrinol 22(12):2596–2608 18945812 10.1210/me.2008-0161 PMC2610364

[14] Li X, Wei W, Huynh H, Zuo H, Wang X, Wan Y (2015) Nur77 prevents excessive osteoclastogenesis by inducing ubiquitin ligase Cbl-b to mediate NFATc1 self-limitation. Elife 4:e07217 26173181 10.7554/eLife.07217 PMC4518709

[15] Sekiya T, Kashiwagi I, Yoshida R, Fukaya T, Morita R, Kimura A et al (2013) Nr4a receptors are essential for thymic regulatory T cell development and immune homeostasis. Nat Immunol 14(3):230–237 23334790 10.1038/ni.2520

[16] Lee MK, Choi H, Gil M, Nikodem VM (2006) Regulation of osteoblast differentiation by Nurr1 in MC3T3-E1 cell line and mouse calvarial osteoblasts. J Cell Biochem 99(3):986–994 16741951 10.1002/jcb.20990

[17] Scholtysek C, Ipseiz N, Böhm C, Krishnacoumar B, Stenzel M, Czerwinski T et al (2018) NR4A1 regulates motility of Osteoclast precursors and serves as target for the modulation of systemic bone turnover. J Bone Min Res 33(11):2035–2047 10.1002/jbmr.3533 29949664

[18] Pirih FQ, Nervina JM, Pham L, Aghaloo T, Tetradis S (2003) Parathyroid hormone induces the nuclear orphan receptor NOR-1 in osteoblasts. Biochem Biophys Res Commun 306(1):144–150 12788080 10.1016/S0006-291X(03)00931-8

[19] Livak KJ, Schmittgen TD (2001) Analysis of relative gene expression data using real-time quantitative PCR and the 2(-Delta Delta C(T)) method. Methods 25(4):402–408 11846609 10.1006/meth.2001.1262

[20] Haller F, Bieg M, Will R, Körner C, Weichenhan D, Bott A et al (2019) Enhancer hijacking activates oncogenic transcription factor NR4A3 in acinic cell carcinomas of the salivary glands. Nat Commun 10(1):368 30664630 10.1038/s41467-018-08069-x PMC6341107

[21] Freise C, Querfeld U (2014) Inhibition of vascular calcification by block of intermediate conductance calcium-activated potassium channels with TRAM-34. Pharmacol Res 85:6–14 24813858 10.1016/j.phrs.2014.04.013

[22] Wei E, Hu M, Wu L, Pan X, Zhu Q, Liu H et al (2024) TGF-β signaling regulates differentiation of MSCs in bone metabolism: disputes among viewpoints. Stem Cell Res Ther 15(1):156 38816830 10.1186/s13287-024-03761-w PMC11140988

[23] Xu P, Liu J, Derynck R (2012) Post-translational regulation of TGF-β receptor and smad signaling. FEBS Lett 586(14):1871–1884 22617150 10.1016/j.febslet.2012.05.010 PMC4240271

[24] Feger M, Hase P, Zhang B, Hirche F, Glosse P, Lang F et al (2017) The production of fibroblast growth factor 23 is controlled by TGF-β2. Sci Rep 7(1):4982 28694529 10.1038/s41598-017-05226-y PMC5503987

[25] Wang Q, Shi W, Lin S, Wang H (2023) FOXO1 regulates osteogenic differentiation of periodontal ligament stem cells through the METTL3 signaling pathway. J Orthop Surg Res 18(1):637 37644500 10.1186/s13018-023-04120-w PMC10463830

[26] Gu K, Fu X, Tian H, Zhang Y, Li A, Wang Y et al (2020) TAZ promotes the proliferation and osteogenic differentiation of human periodontal ligament stem cells via the p-SMAD3. J Cell Biochem 121(2):1101–1113 31478222 10.1002/jcb.29346

[27] Liu F, Wang X, Zheng B, Li D, Chen C, Lee IS et al (2020) USF2 enhances the osteogenic differentiation of PDLCs by promoting ATF4 transcriptional activities. J Periodontal Res 55(1):68–76 31448831 10.1111/jre.12689

[28] Freire PR, Conneely OM (2018) NR4A1 and NR4A3 restrict HSC proliferation via reciprocal regulation of C/EBPα and inflammatory signaling. Blood 131(10):1081–1093 29343483 10.1182/blood-2017-07-795757 PMC5863701

[29] Lin SC, Yao CY, Hsu CA, Lin CT, Calkins MJ, Kuo YY et al (2022) Functional association of NR4A3 downregulation with impaired differentiation in myeloid leukemogenesis. Ann Hematol 101(10):2209–2218 36040481 10.1007/s00277-022-04961-1 PMC9463347

[30] Pirih FQ, Tang A, Ozkurt IC, Nervina JM, Tetradis S (2004) Nuclear orphan receptor Nurr1 directly transactivates the osteocalcin gene in osteoblasts. J Biol Chem 279(51):53167–53174 15485875 10.1074/jbc.M405677200

[31] Lammi J, Huppunen J, Aarnisalo P (2004) Regulation of the osteopontin gene by the orphan nuclear receptor NURR1 in osteoblasts. Mol Endocrinol 18(6):1546–1557 14988426 10.1210/me.2003-0247

[32] Voraberger B, Mayr JA, Fratzl-Zelman N, Blouin S, Uday S, Kopajtich R et al (2023) Investigating the role of ASCC1 in the causation of bone fragility. Front Endocrinol (Lausanne) 14:1137573 37455927 10.3389/fendo.2023.1137573 PMC10348481

[33] Pearen MA, Ryall JG, Maxwell MA, Ohkura N, Lynch GS, Muscat GE (2006) The orphan nuclear receptor, NOR-1, is a target of beta-adrenergic signaling in skeletal muscle. Endocrinology 147(11):5217–5227 16901967 10.1210/en.2006-0447

[34] Kumar N, Wang H, Liu D, Collins S (2009) Liver X receptor is a regulator of orphan nuclear receptor NOR-1 gene transcription in adipocytes. Int J Obes (Lond) 33(5):519–524 19238156 10.1038/ijo.2009.32

[35] Veum VL, Dankel SN, Gjerde J, Nielsen HJ, Solsvik MH, Haugen C et al (2012) The nuclear receptors NUR77, NURR1 and NOR1 in obesity and during fat loss. Int J Obes (Lond) 36(9):1195–1202 22143616 10.1038/ijo.2011.240

[36] Tang Y, Wu X, Lei W, Pang L, Wan C, Shi Z et al (2009) TGF-beta1-induced migration of bone mesenchymal stem cells couples bone resorption with formation. Nat Med 15(7):757–765 19584867 10.1038/nm.1979 PMC2727637

[37] Seo HS, Serra R (2009) Tgfbr2 is required for development of the skull vault. Dev Biol 334(2):481–490 19699732 10.1016/j.ydbio.2009.08.015 PMC2753698

[38] Peters SB, Wang Y, Serra R (2017) Tgfbr2 is required in osterix expressing cells for postnatal skeletal development. Bone 97:54–64 28043895 10.1016/j.bone.2016.12.017 PMC5368008

[39] Jann J, Gascon S, Roux S, Faucheux N (2020) Influence of the TGF-β superfamily on Osteoclasts/Osteoblasts balance in physiological and pathological bone conditions. Int J Mol Sci 21(20) 10.3390/ijms21207597 PMC7589189 33066607

[40] Hong S, Lazerka N, Jeon BJ, Kim JD, Erdenebileg S, Nho CW et al (2024) Osteogenic effects of the Diospyros lotus L. Leaf Extract on MC3T3-E1 pre-osteoblasts and ovariectomized mice via BMP2/4 and TGF β pathways. Nutrients 16(8) 10.3390/nu16081247 PMC11053699 38674937

[41] Li S, Siengdee P, Oster M, Reyer H, Wimmers K, Ponsuksili S (2023) Transcriptome changes during osteogenesis of porcine mesenchymal stem cells derived from different types of synovial membranes and genetic background. Sci Rep 13(1):10048 37344635 10.1038/s41598-023-37260-4 PMC10284927

[42] Matsunobu T, Torigoe K, Ishikawa M, de Vega S, Kulkarni AB, Iwamoto Y et al (2009) Critical roles of the TGF-beta type I receptor ALK5 in perichondrial formation and function, cartilage integrity, and osteoblast differentiation during growth plate development. Dev Biol 332(2):325–338 19501582 10.1016/j.ydbio.2009.06.002 PMC2716725

[43] Almuraikhi N (2023) Inhibition of TGF-β type I receptor by SB505124 down-regulates osteoblast differentiation and mineralization of human mesenchymal stem cells. Cell Biochem Funct 41(5):564–572 37232472 10.1002/cbf.3812

[44] Tian M, Han YB, Yang GY, Li JL, Shi CS, Tian D (2023) The role of lactoferrin in bone remodeling: evaluation of its potential in targeted delivery and treatment of metabolic bone diseases and orthopedic conditions. Front Endocrinol (Lausanne) 14:1218148 37680888 10.3389/fendo.2023.1218148 PMC10482240

[45] Baron R, Kneissel M (2013) WNT signaling in bone homeostasis and disease: from human mutations to treatments. Nat Med 19(2):179–192 23389618 10.1038/nm.3074

[46] Li K, Liu L, Liu H, Liu Y, Xing J, Song J et al (2024) Hippo/YAP1 promotes osteoporotic mice bone defect repair via the activating of wnt signaling pathway. Cell Signal 116:111037 38184268 10.1016/j.cellsig.2024.111037

[47] Luo K (2017) Signaling Cross talk between TGF-β/Smad and other Signaling pathways. Cold Spring Harb Perspect Biol 9(1) 10.1101/cshperspect.a022137 PMC5204325 27836834

