# Supplementary material for: NOR1 promotes the osteoblastic differentiation of human periodontal ligament stem cells via TGF-β signaling pathway
Source: Cell Mol Life Sci. 2024 Aug 9;81(1):338. doi: 10.1007/s00018-024-05356-3 (PMC11335260; doi:10.1007/s00018-024-05356-3)
Supplement: Supplementary file 1 — Supplementary Material 1 [file 18_2024_5356_MOESM1_ESM.docx]

**Supplementary Table 1**. Primer sequences for CHIP-qPCR

| Name | Forward primer(5’-3’) | Reverse primer(5’-3’) |
| --- | --- | --- |
| CHIP-1 | TAAGGGGCCCAAGACAAGTG | TGCTGCTCTATTTGCAATGTC |
| CHIP-2 | GAGGGCAAATTGGGACTGGA | GAGGCCTGCAACTTGCTCTA |
| CHIP-3 | GTGTCCTAGACAGGAGGGAGA | AAGGCTTCCTTCCGGGAGAT |
| CHIP-4 | ACGTAACAGCCTCCTTGGCT | CTCTGCGACGCCAAAATGA |
| CHIP-5 | GCGTCTCGCAGTAAATTAGGA | GTTCCCCCTTCTTAGCACCC |
| CHIP-6 | CTGGATCGGGAAGGGGTTTG | ACACACCCCCAAATAGCCTC |
| CHIP-7 | CTTCGGGGGAATGCTGAAGT | CATTCACAGCTCCGCATTGG |
