# Supplementary material for: NOR1 promotes the osteoblastic differentiation of human periodontal ligament stem cells via TGF-β signaling pathway
Source: Cell Mol Life Sci. 2024 Aug 9;81(1):338. doi: 10.1007/s00018-024-05356-3 (PMC11335260; doi:10.1007/s00018-024-05356-3)
Supplement: Supplementary file 2 — Supplementary Material 2 [file 18_2024_5356_MOESM2_ESM.docx]

**Materials and Methods**

*Flow cytometry*

hPDLSCs were digested by tyrisin and counted by cell counter, followed by centrifuged with 1200 rpm for 5 min. The supernatant was discared, and the cell sediment was resuspended with PBS containing 2% FBS. Then, the cell suspension was divided into 150 μl per tube and incubated with FITC-labeled antibody against CD44, CD105, HLA-DR, CD45, CD34, CD146 and CD90 ﻿(1 : 10; BD Biosciences, Franklin Lakes, ﻿NJ, USA). Cells incubated with PBS were used as negative control. After incubation for 1 h in 4℃, cell suspension was washed with PBS containing 2% FBS for three times, and centrifuged with 1200 rpm for 5 min. Subsequently, the cell deposit was re-suspended with 350 μl PBS containing 2% FBS and then the flow cytometry was applied to detect the expression of the surface markers.

*Immunofluorescence*

PDLSCs were seeded on confocal dish with the density of 5 x 10^5^ cells/dish. After sub-confluence, the culture medium was discarded and cells were rinsed with PBS for three times, followed by being fixed with 4% paraformaldehyde for 15 min. Then, cells were permeabilized with 0.25% Triton X-100 for 15 min and blocked with bovine serum albumin for 1 hour. Subsequently, PDLSCs were incubated with primary antibody against vimentin (1:100, Boster Biological Technology, Wuhan, China) or pan cytokeratin (PCK, 1:100, Boster Biological Technology, Wuhan, China) at 4℃ overnight. Cells incubated with PBS were used as negative control. Next day, cells were incubated with fluorescin isothiocyanate-conjugated secondary antibody ﻿(1:200; Proteintech Group, Inc., Rosemont, USA) for 1 hour at room temperature after washing. Lastly, cells were stained with Dapi and observed under a confocal microscope.

Adipogenic differentiation

PDLSCs were cultured in six well plate. After reaching to sub-confluence, cells were treated with adipogenic differentiation medium according to the manufacture’s instructions (Cyagen, Suzhou, China). After 18 d, Oil red O staining was used to observe lipid accumulation.
