# Supplementary material for: NOR1 promotes the osteoblastic differentiation of human periodontal ligament stem cells via TGF-β signaling pathway
Source: Cell Mol Life Sci. 2024 Aug 9;81(1):338. doi: 10.1007/s00018-024-05356-3 (PMC11335260; doi:10.1007/s00018-024-05356-3)

| Gene | The size of product | The annealing temperature | GenBank Accession numbers |
| --- | --- | --- | --- |
| ALPL | 123 bp | 60 ℃ | [NM_000478.6](https://www.ncbi.nlm.nih.gov/entrez/viewer.fcgi?db=nucleotide&id=1519315936)  [NM_001369803.2](https://www.ncbi.nlm.nih.gov/entrez/viewer.fcgi?db=nucleotide&id=1676440594)  [NM_001369805.2](https://www.ncbi.nlm.nih.gov/entrez/viewer.fcgi?db=nucleotide&id=1675178461)  [NM_001369804.2](https://www.ncbi.nlm.nih.gov/entrez/viewer.fcgi?db=nucleotide&id=1674986265)  [NM_001127501.4](https://www.ncbi.nlm.nih.gov/entrez/viewer.fcgi?db=nucleotide&id=1676441661) |
| RUNX2 | 166 bp | 60 ℃ | [NM_001015051.4](https://www.ncbi.nlm.nih.gov/entrez/viewer.fcgi?db=nucleotide&id=1890358904)  [NM_001278478.2](https://www.ncbi.nlm.nih.gov/entrez/viewer.fcgi?db=nucleotide&id=1609559059) |
| COL1A1 | 170 bp | 60 ℃ | [NM_000088.4](https://www.ncbi.nlm.nih.gov/entrez/viewer.fcgi?db=nucleotide&id=1777425449) |
| OCN | 117 bp | 60 ℃ | [NM_199173.6](https://www.ncbi.nlm.nih.gov/entrez/viewer.fcgi?db=nucleotide&id=1519313061) |
| NOR1 | 110 bp | 60 ℃ | [NM_173200.3](https://www.ncbi.nlm.nih.gov/entrez/viewer.fcgi?db=nucleotide&id=1890259025)  [NM_006981.4](https://www.ncbi.nlm.nih.gov/entrez/viewer.fcgi?db=nucleotide&id=1519243370) |
| TGFBR1 | 121 bp | 60 ℃ | [NM_001130916.3](https://www.ncbi.nlm.nih.gov/entrez/viewer.fcgi?db=nucleotide&id=1675081664) |
| β-actin | 78 bp | 60 ℃ | [NM_001101.5](https://www.ncbi.nlm.nih.gov/entrez/viewer.fcgi?db=nucleotide&id=1519311456) |

The results of Primer-BLAST

1.ALPL:


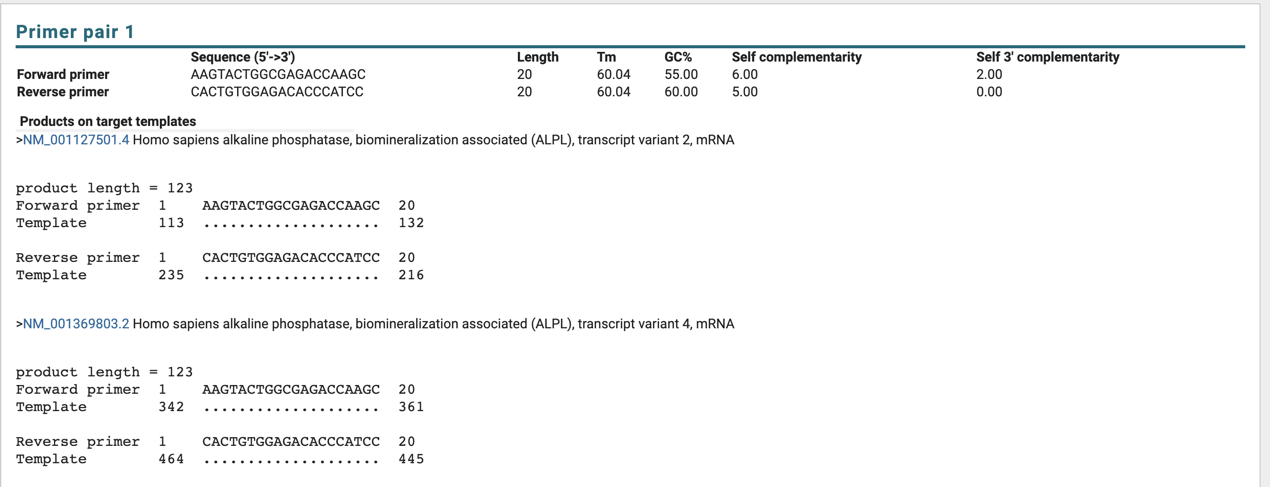


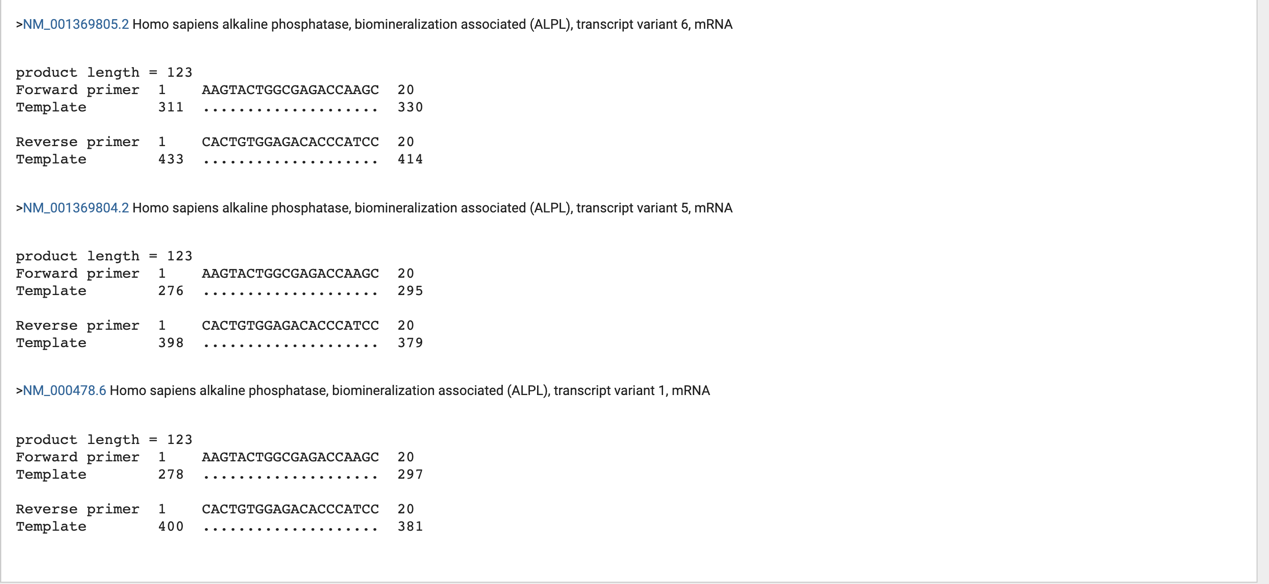


2.RUNX2:


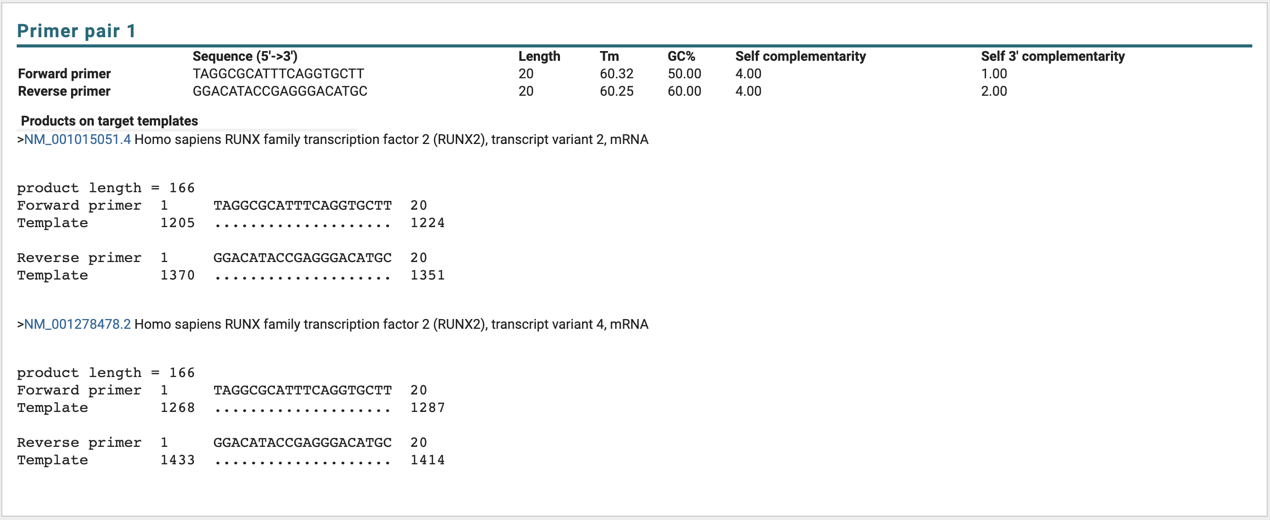


3. COL1A1:


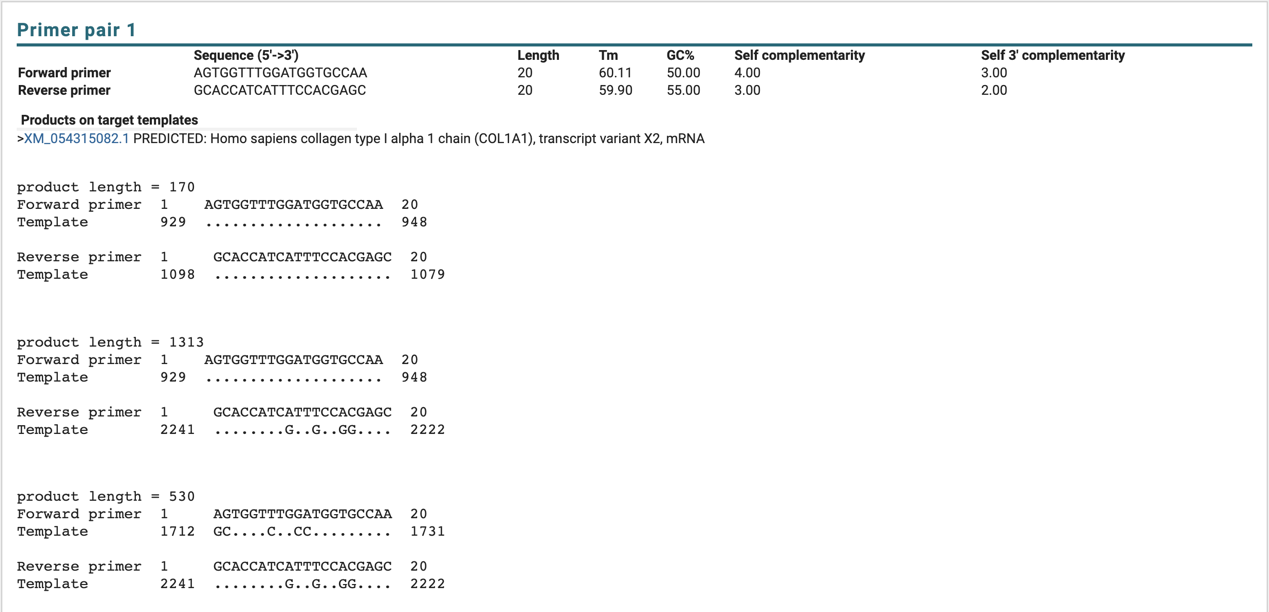


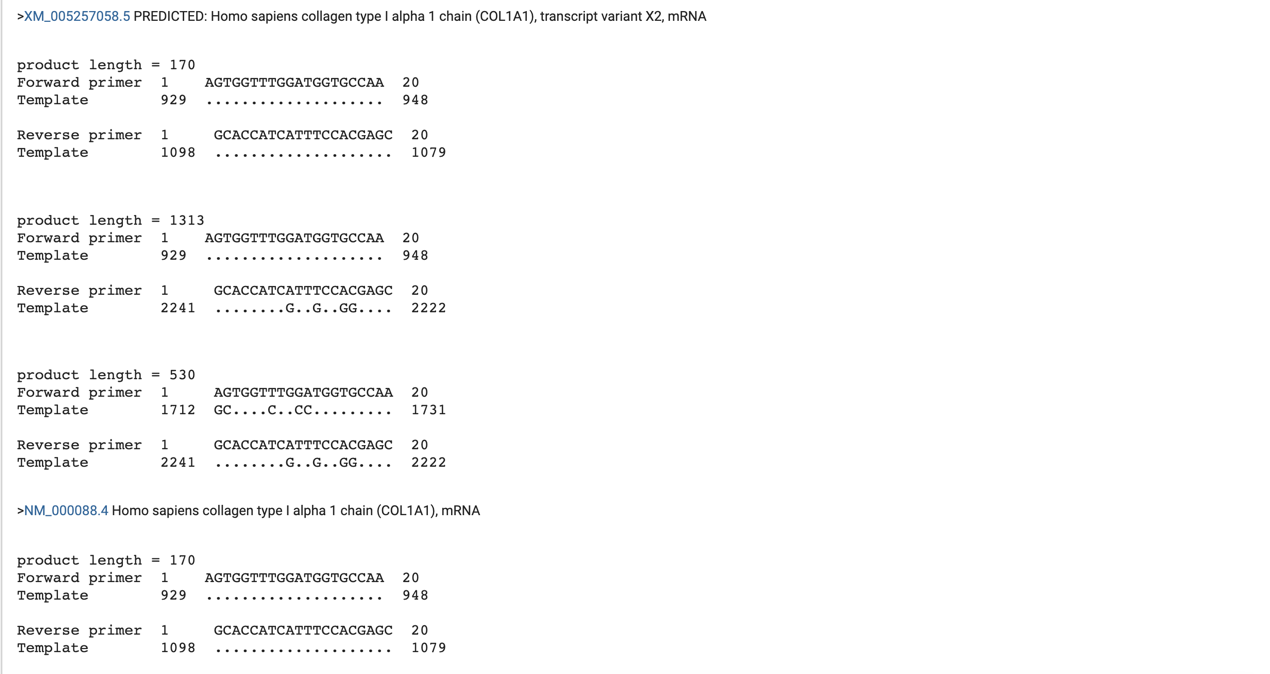


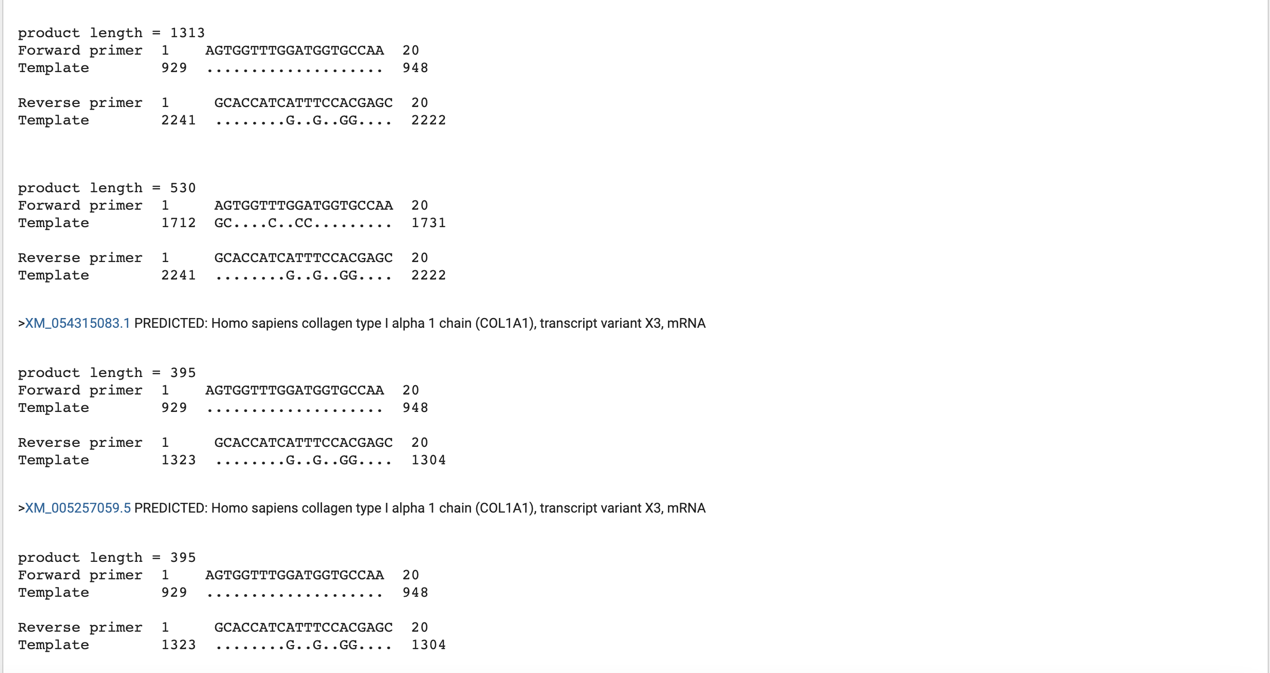


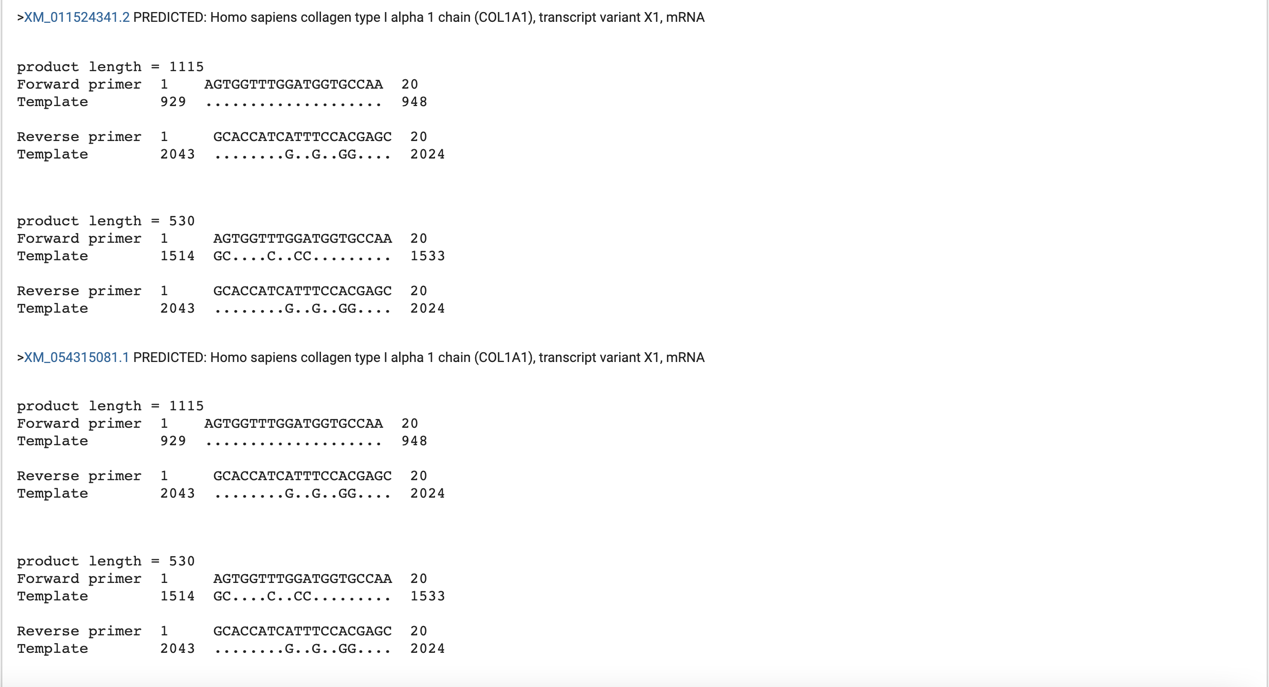


4.OCN(BGLAP):


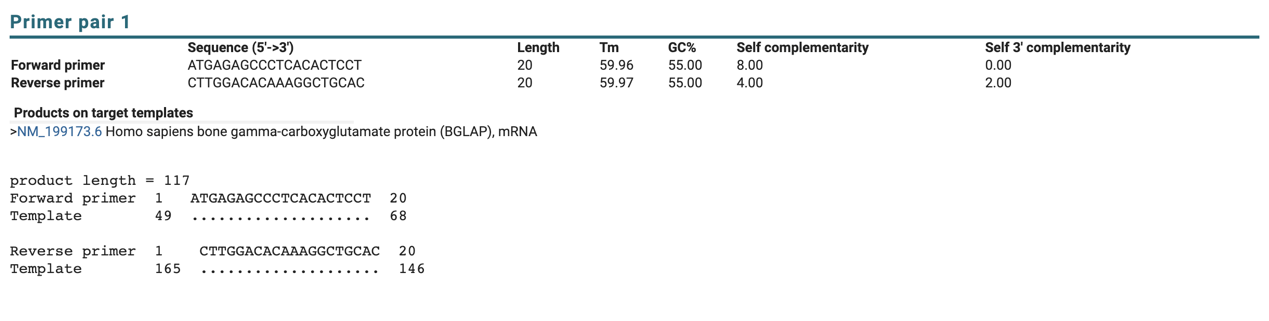


5.NOR1:


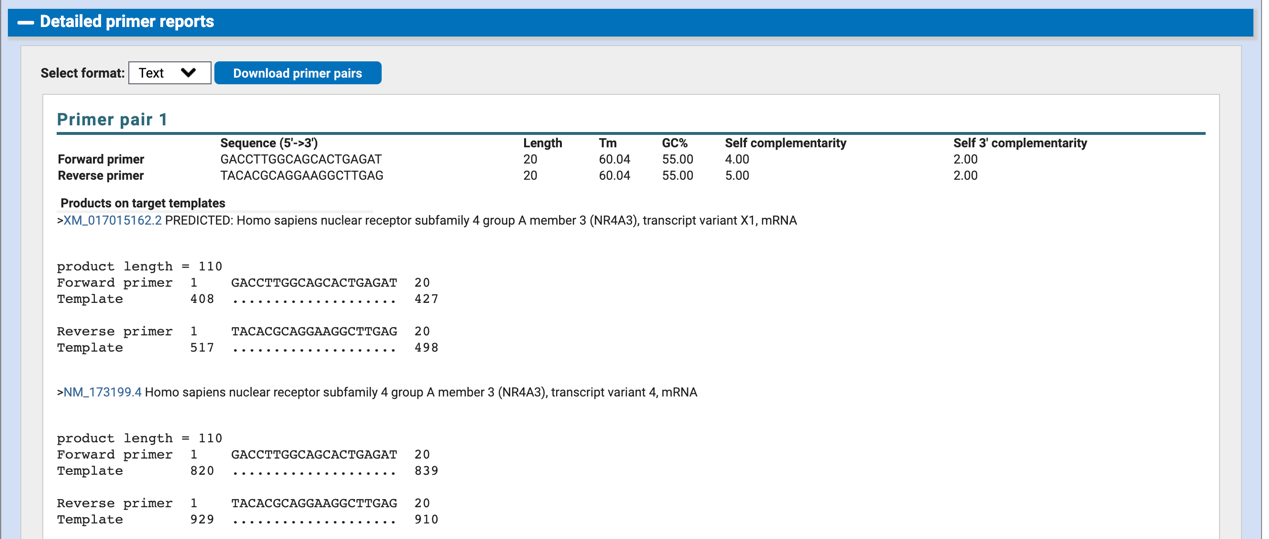


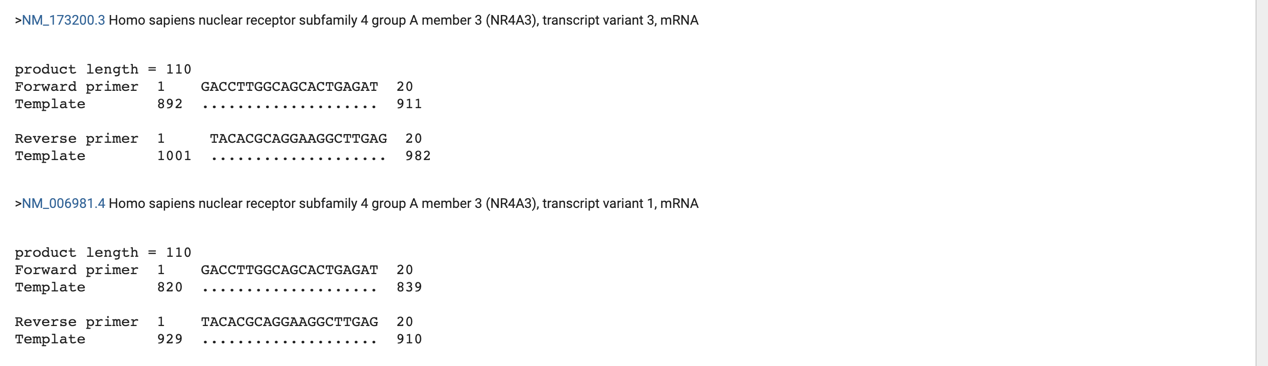


6.TGFBR1:


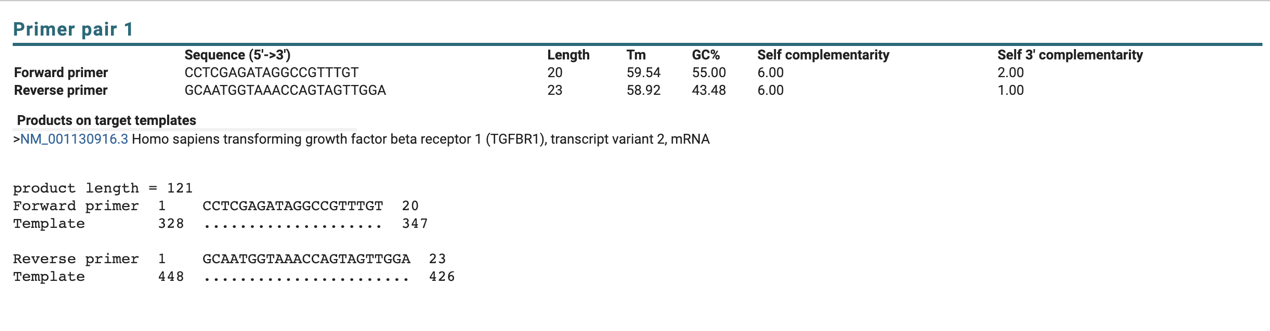


7. β-actin:


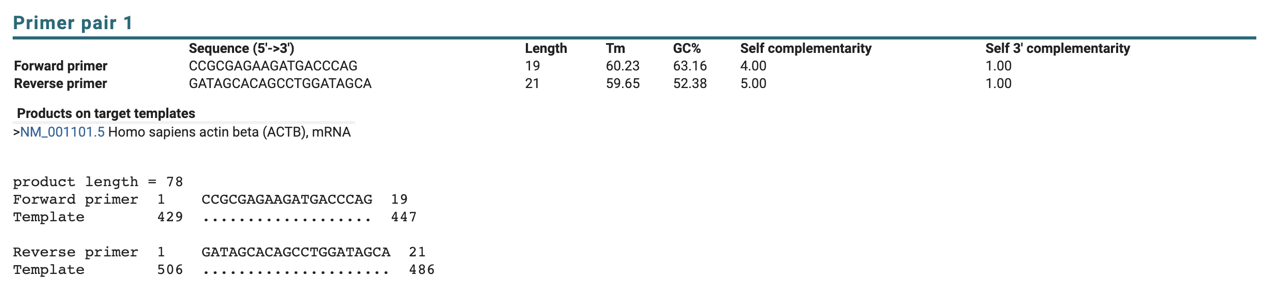

Supplement: Supplementary file 3 — Supplementary Material 3 [file 18_2024_5356_MOESM3_ESM.docx]
