# Supplementary material for: NOR1 promotes the osteoblastic differentiation of human periodontal ligament stem cells via TGF-β signaling pathway
Source: Cell Mol Life Sci. 2024 Aug 9;81(1):338. doi: 10.1007/s00018-024-05356-3 (PMC11335260; doi:10.1007/s00018-024-05356-3)
Supplement: Supplementary file 4 — Supplementary Material 4 [file 18_2024_5356_MOESM4_ESM.docx]

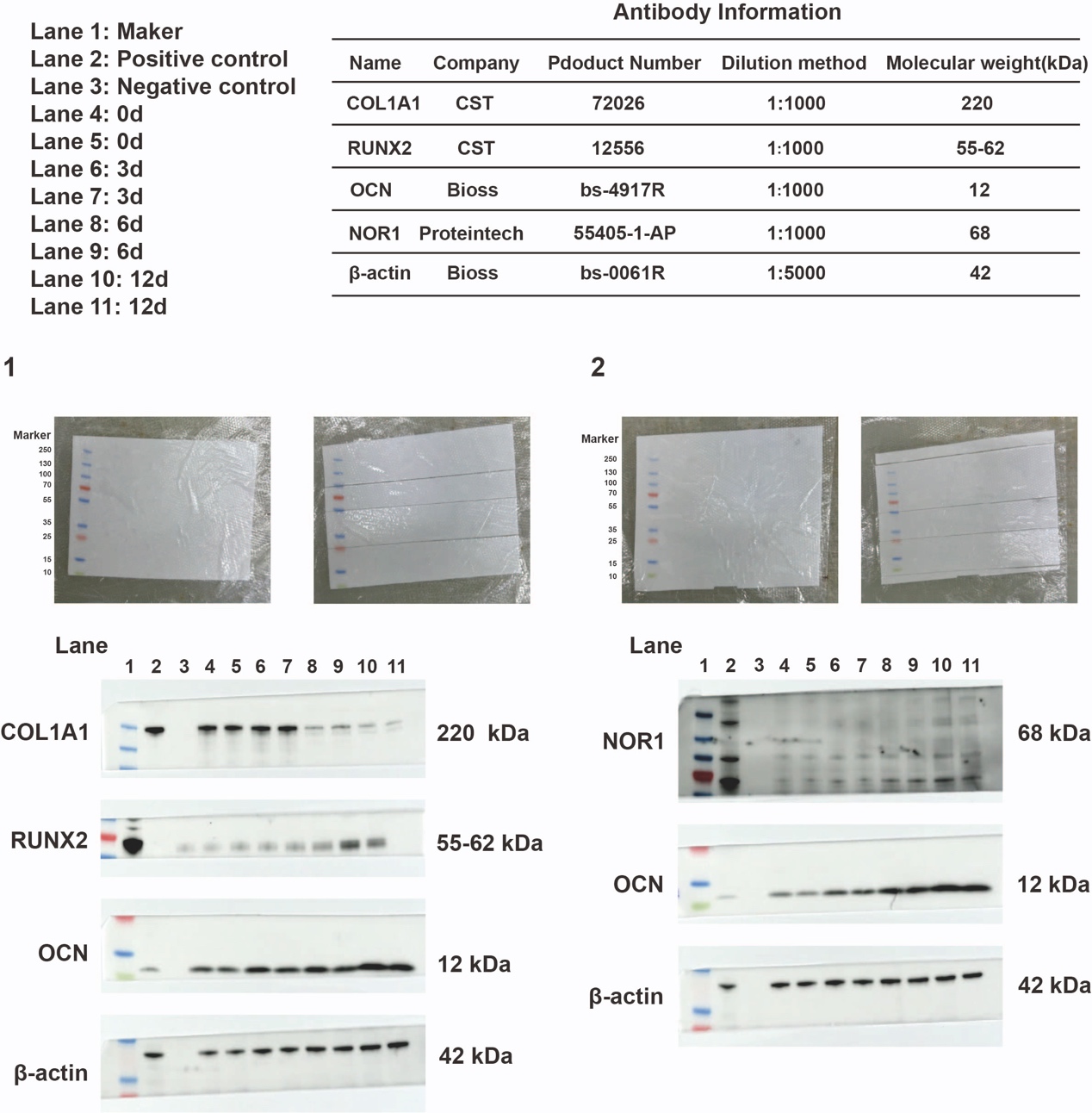


**Figure 1.** NOR1 is upregulated during osteogenic differentiation of human PDLSCs. ﻿Each protein sample was loaded on SDS-PAGE gel as above order. The amount of loading protein is 40 μg for each group. The samples loading in the left membrane are the same with that of the right membrane. β-actin was used as internal control. The antibody information used in these experiments have been listed in the above table. The protein bands were visualized using an enhanced chemiluminescence (ECL) kit (Millipore, Billerica, MA, USA) and captured using Tanon 5200 chemiluminescent imaging system (shanghai).


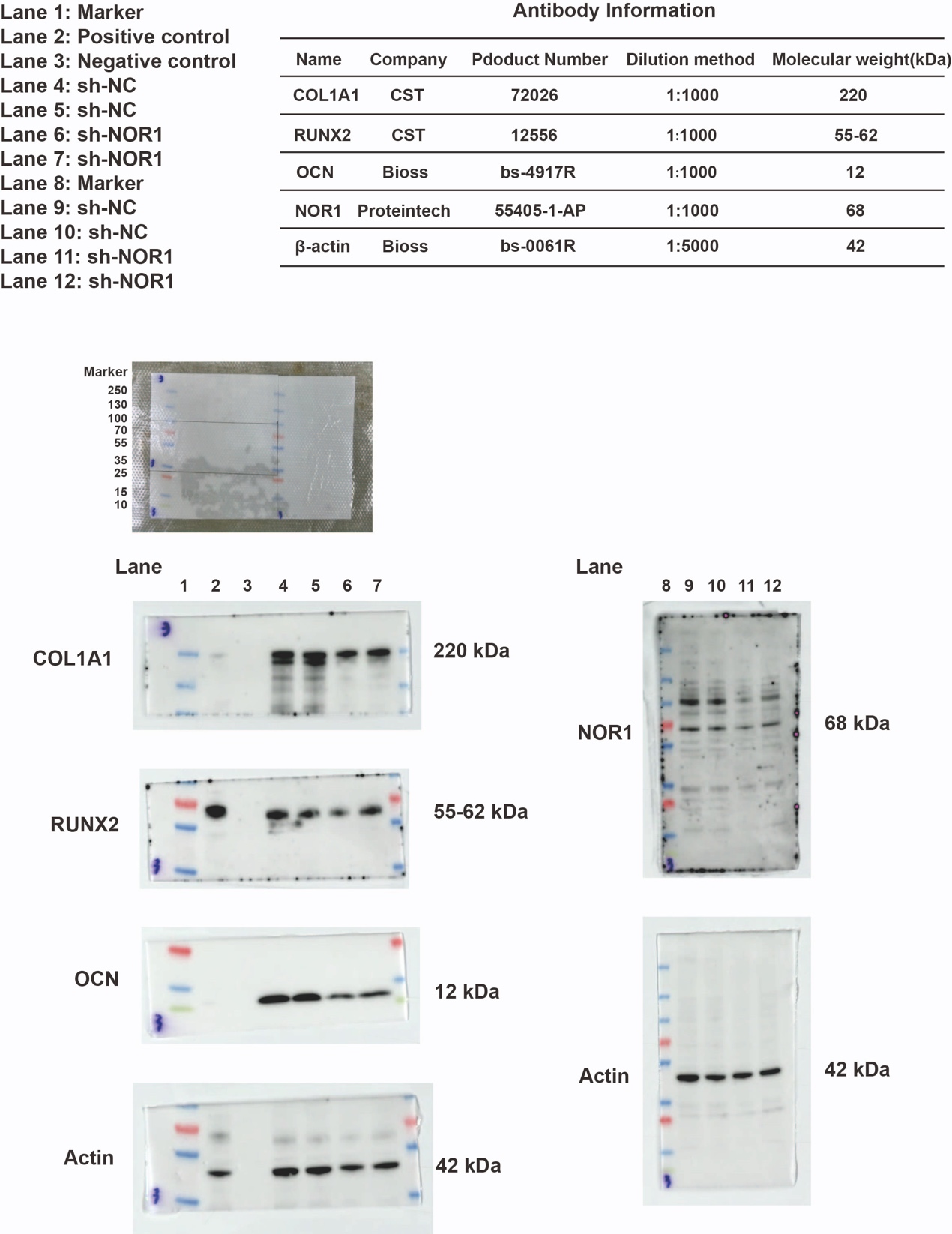


**Figure 2.** Knockdown of NOR1 inhibits the osteogenic differentiation potential of PDLSCs. Each protein sample was loaded on SDS-PAGE gel as above order. The amount of loading protein is 40 μg for each group. The samples of lane 4, 5, 6, 7 are the same with that of lane 9, 10, 11, 12. β-actin was used as internal control. The antibody information used in these experiments have been listed in the above table. The protein bands were visualized using an enhanced chemiluminescence (ECL) kit (Millipore, Billerica, MA, USA) and captured using Tanon 5200 chemiluminescent imaging system (shanghai).


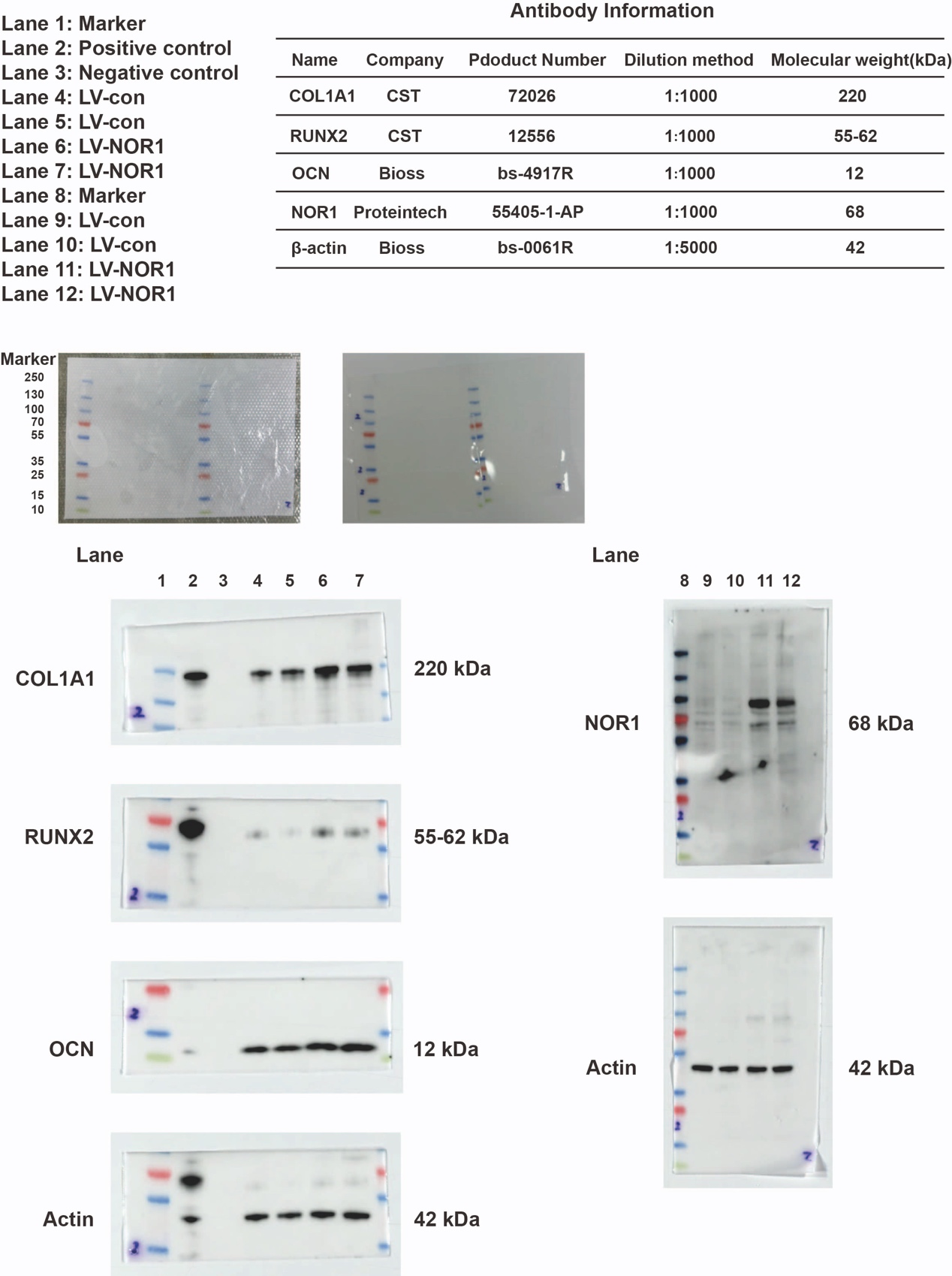


**Figure 3.** NOR1 overexpression promotes the osteoblast differentiation of PDLSCs. Each protein sample was loaded on SDS-PAGE gel as above order. The amount of loading protein is 40 μg for each group. The samples of lane 4, 5, 6, 7 are the same with that of lane 9, 10, 11, 12. β-actin was used as internal control. The antibody information used in these experiments have been listed in the above table. The protein bands were visualized using an enhanced chemiluminescence (ECL) kit (Millipore, Billerica, MA, USA) and captured using Tanon 5200 chemiluminescent imaging system (shanghai).


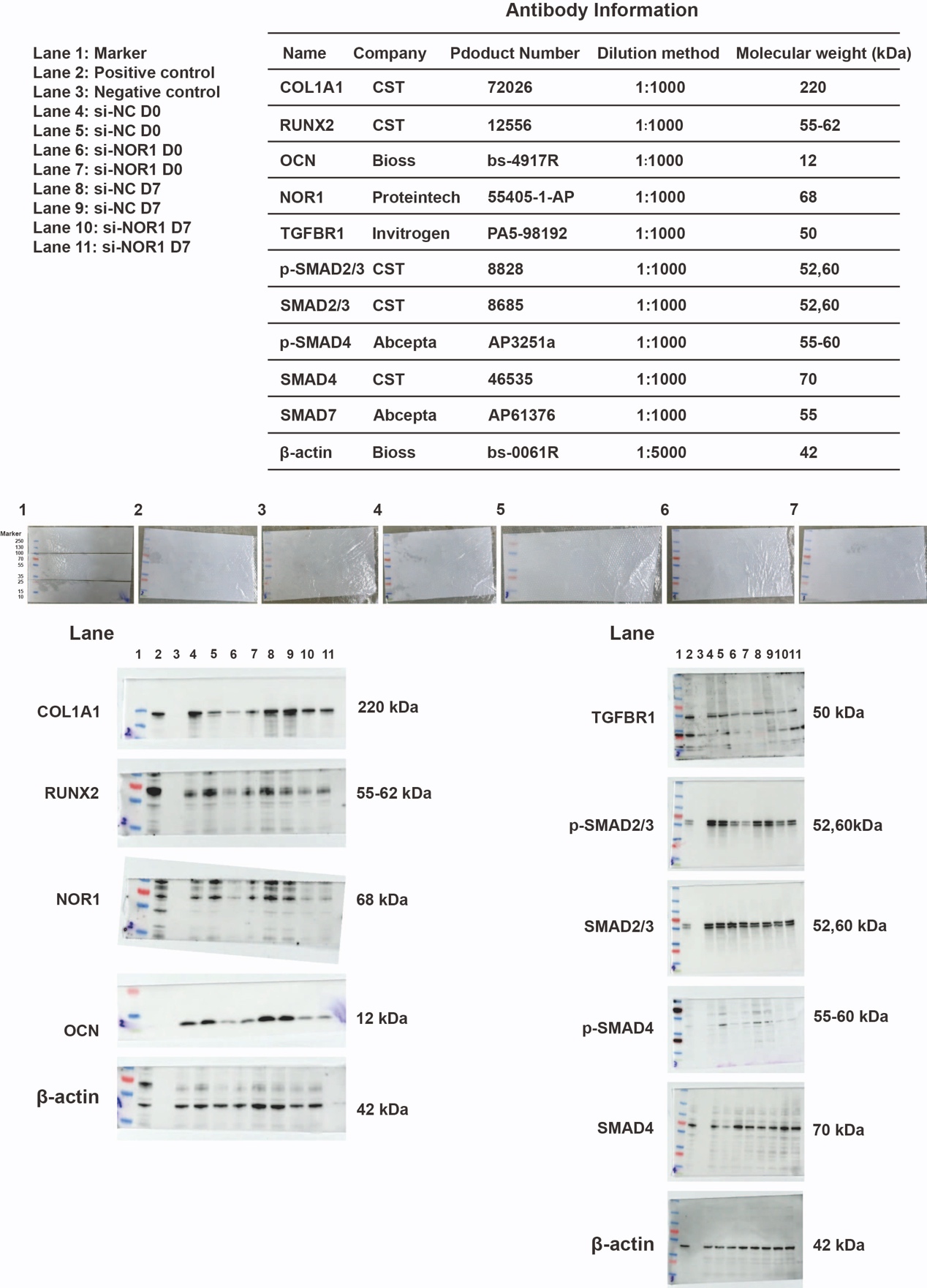


**Figure 4.** NOR1 knockdown inhibits the activation of TGF-β/SMAD signaling pathway during osteoblast differentiation of PDLSCs. Each protein sample was loaded on SDS-PAGE gel as above order. The amount of loading protein is 40 μg for each group. The samples loading in the left membrane(1) are the same with that of the right membranes(2-7). β-actin was used as internal control. The antibody information used in these experiments have been listed in the above table. The protein bands were visualized using an enhanced chemiluminescence (ECL) kit (Millipore, Billerica, MA, USA) and captured using Tanon 5200 chemiluminescent imaging system (shanghai).


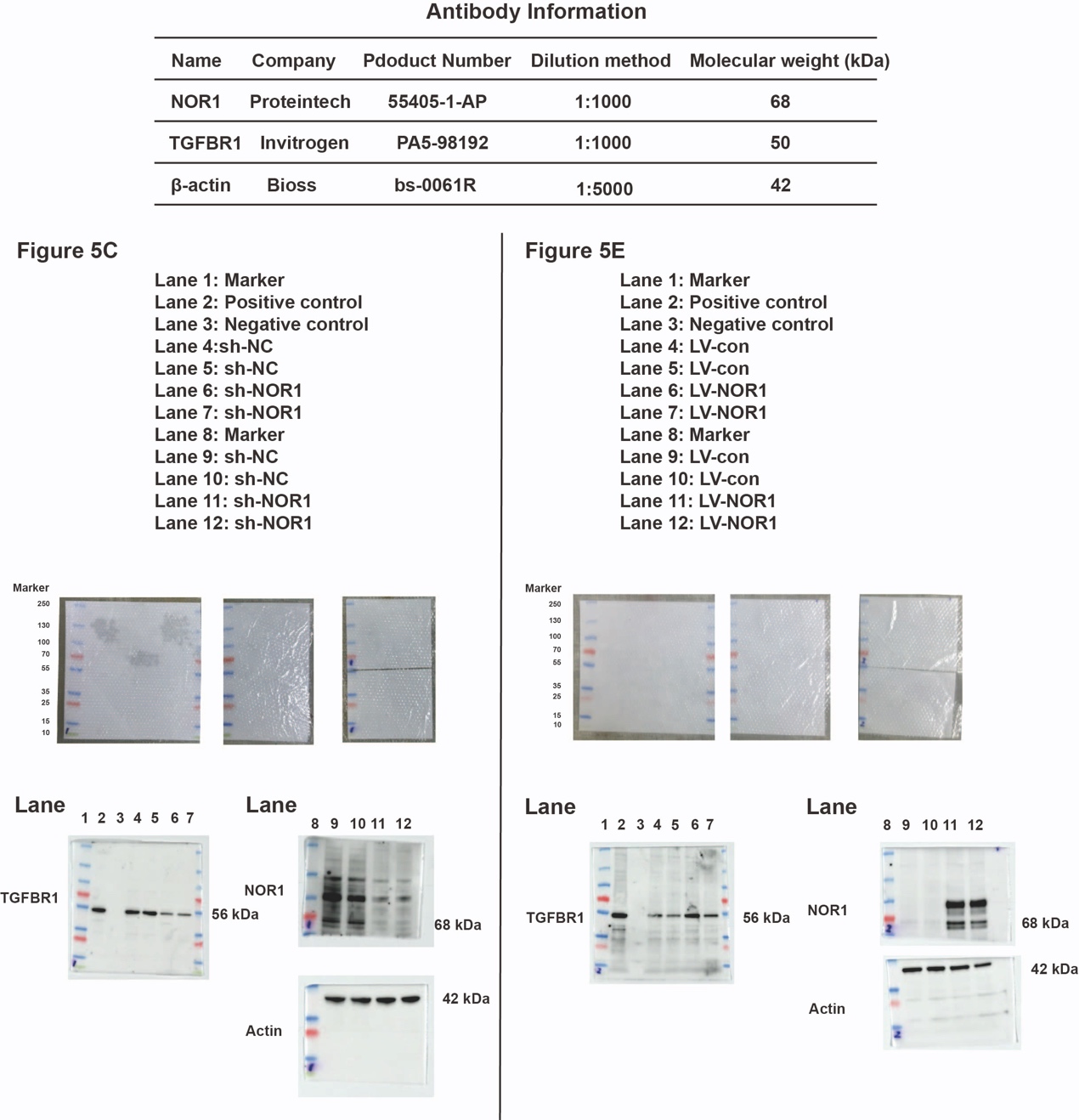


**Figure 5.** NOR1 regulates the expression of TGFBR1 detected by Western blot. Each protein sample was loaded on SDS-PAGE gel as above order. The amount of loading protein is 40 μg for each group. For the left gel(Figure 5C): The samples of lane 4, 5, 6, 7 are the same with that of lane 9, 10, 11, 12. For the right gel(Figure 5E): The samples of lane 4, 5, 6, 7 are the same with that of lane 9, 10, 11, 12. β-actin was used as internal control. The antibody information used in these experiments have been listed in the above table. The protein bands were visualized using an enhanced chemiluminescence (ECL) kit (Millipore, Billerica, MA, USA) and captured using Tanon 5200 chemiluminescent imaging system (shanghai).


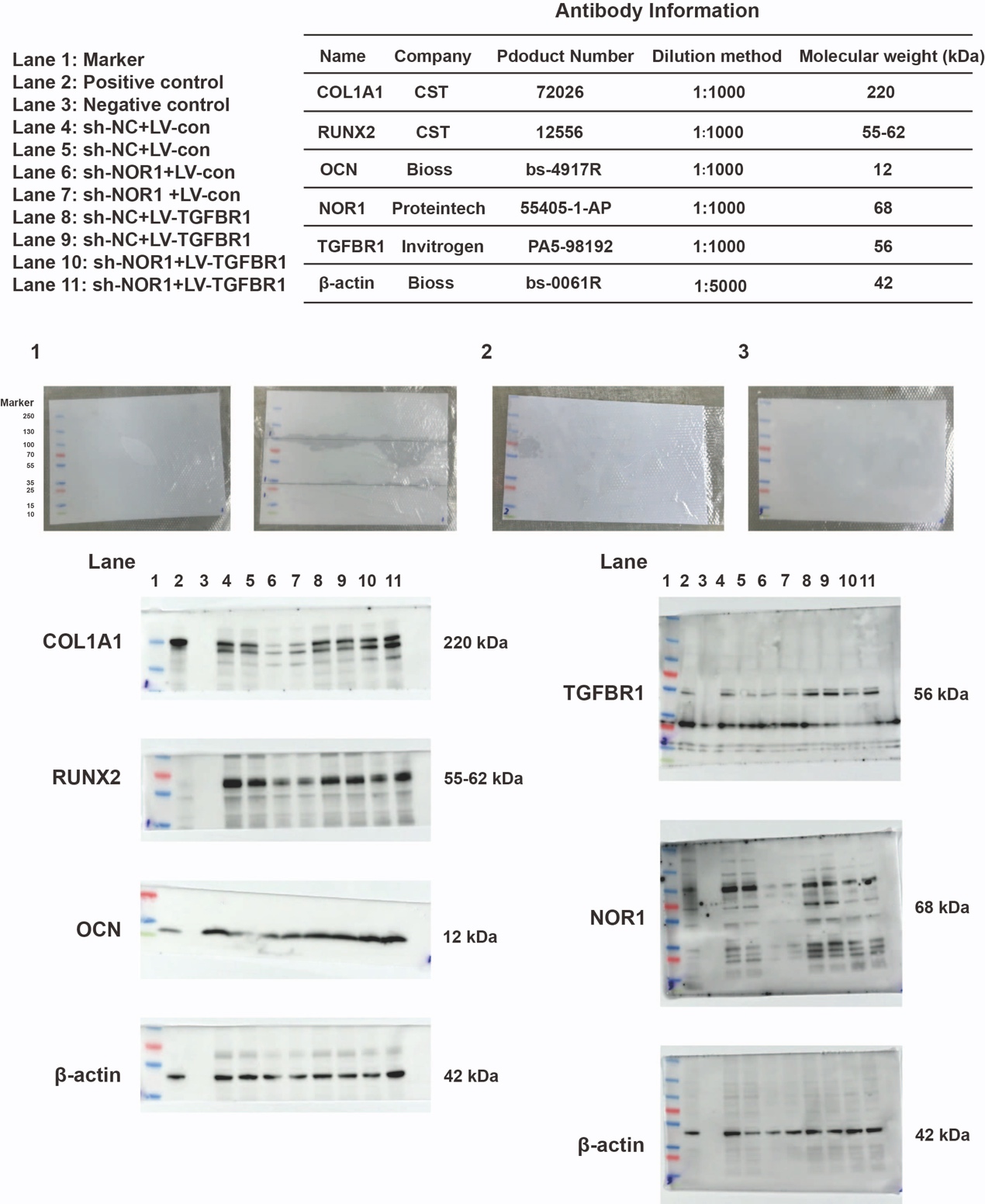


**Figure 6.** NOR1 modulates osteogenesis of PDLSCs through targeting TGFBR1 directly. Each protein sample was loaded on SDS-PAGE gel as above order. The amount of loading protein is 40 μg for each group. The samples loading in the left membrane(1) are the same with that of the right membrane(2-3). β-actin was used as internal control. The antibody information used in these experiments have been listed in the above table. The protein bands were visualized using an enhanced chemiluminescence (ECL) kit (Millipore, Billerica, MA, USA) and captured using Tanon 5200 chemiluminescent imaging system (shanghai).


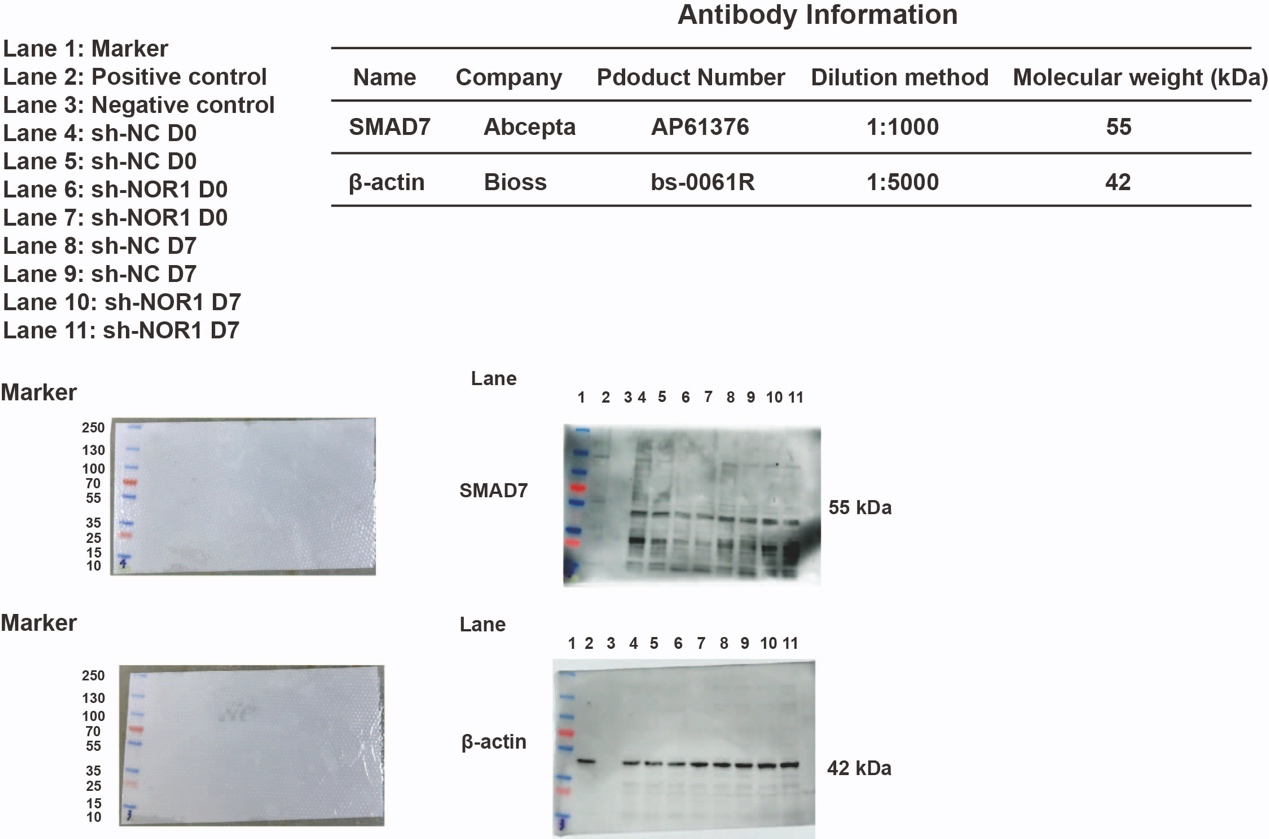


**Supplementary Figure 2.** The expression of SMAD7 in PDLSCs transfected with sh-NOR1 with or without osteogenic induction detected by western blot. Each protein sample was loaded on SDS-PAGE gel as above order. The amount of loading protein is 40 μg for each group. The samples loading in the upper membrane are the same with that of the lower membrane. Also, these protein samples used are the same with that of Figure 4. β-actin was used as internal control. The antibody information used in these experiments have been listed in the above table. The protein bands were visualized using an enhanced chemiluminescence (ECL) kit (Millipore, Billerica, MA, USA) and captured using Tanon 5200 chemiluminescent imaging system (shanghai).
