# Supplementary figures and images for: NOR1 promotes the osteoblastic differentiation of human periodontal ligament stem cells via TGF-β signaling pathway
Source: Cell Mol Life Sci. 2024 Aug 9;81(1):338. doi: 10.1007/s00018-024-05356-3 (PMC11335260; doi:10.1007/s00018-024-05356-3)

**Shapiro-Wilk test for the normal distribution of data**


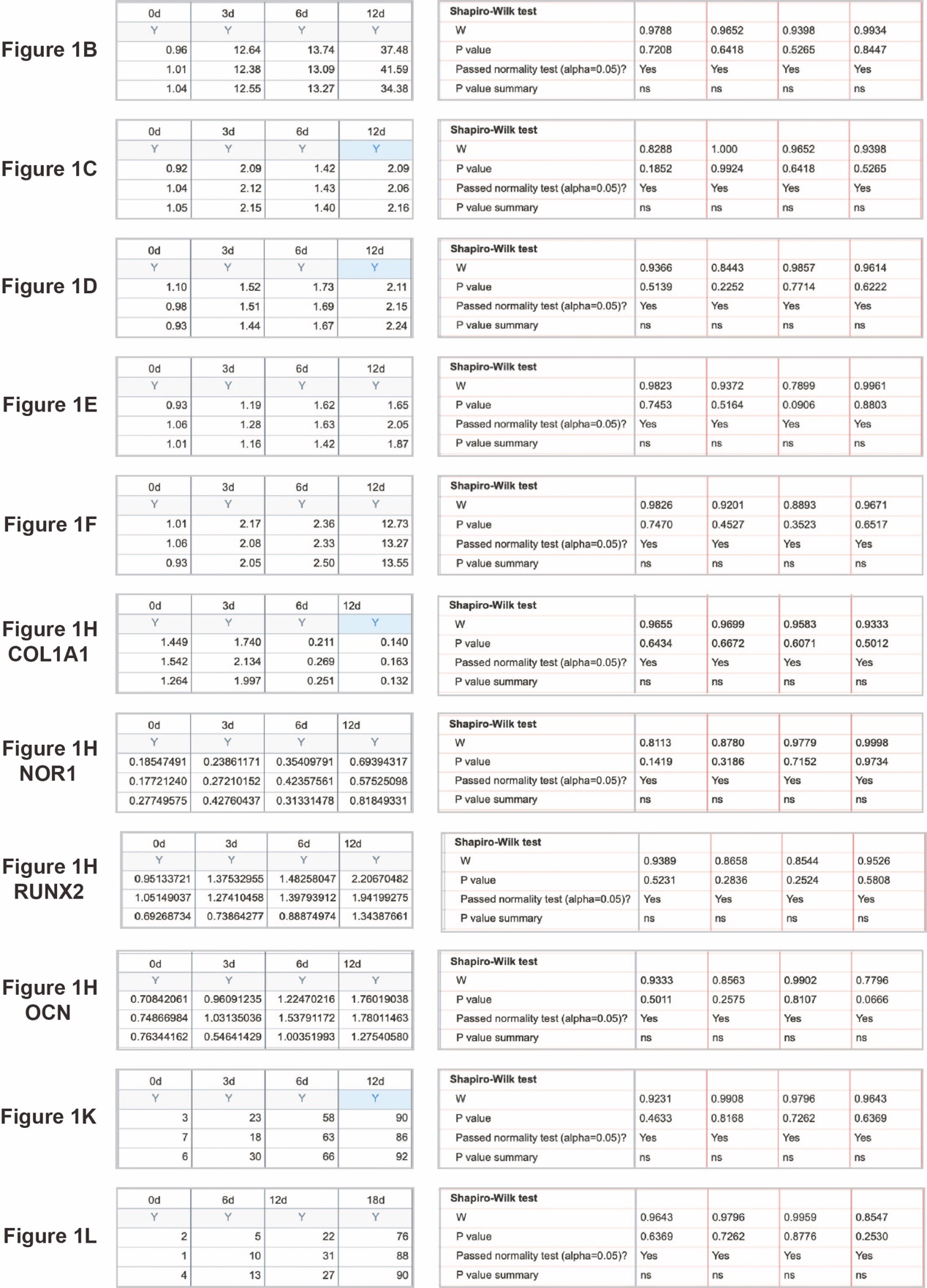


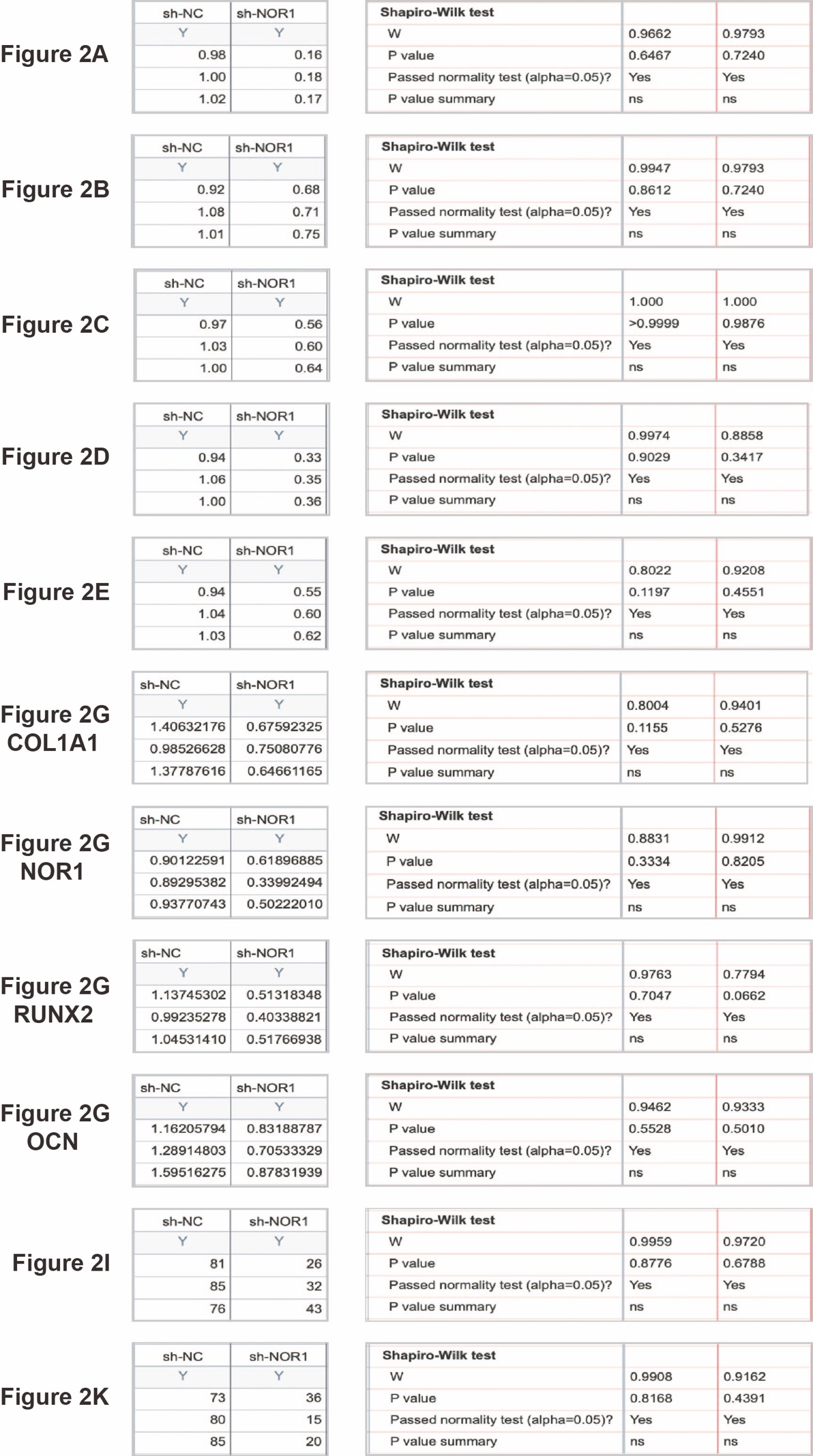


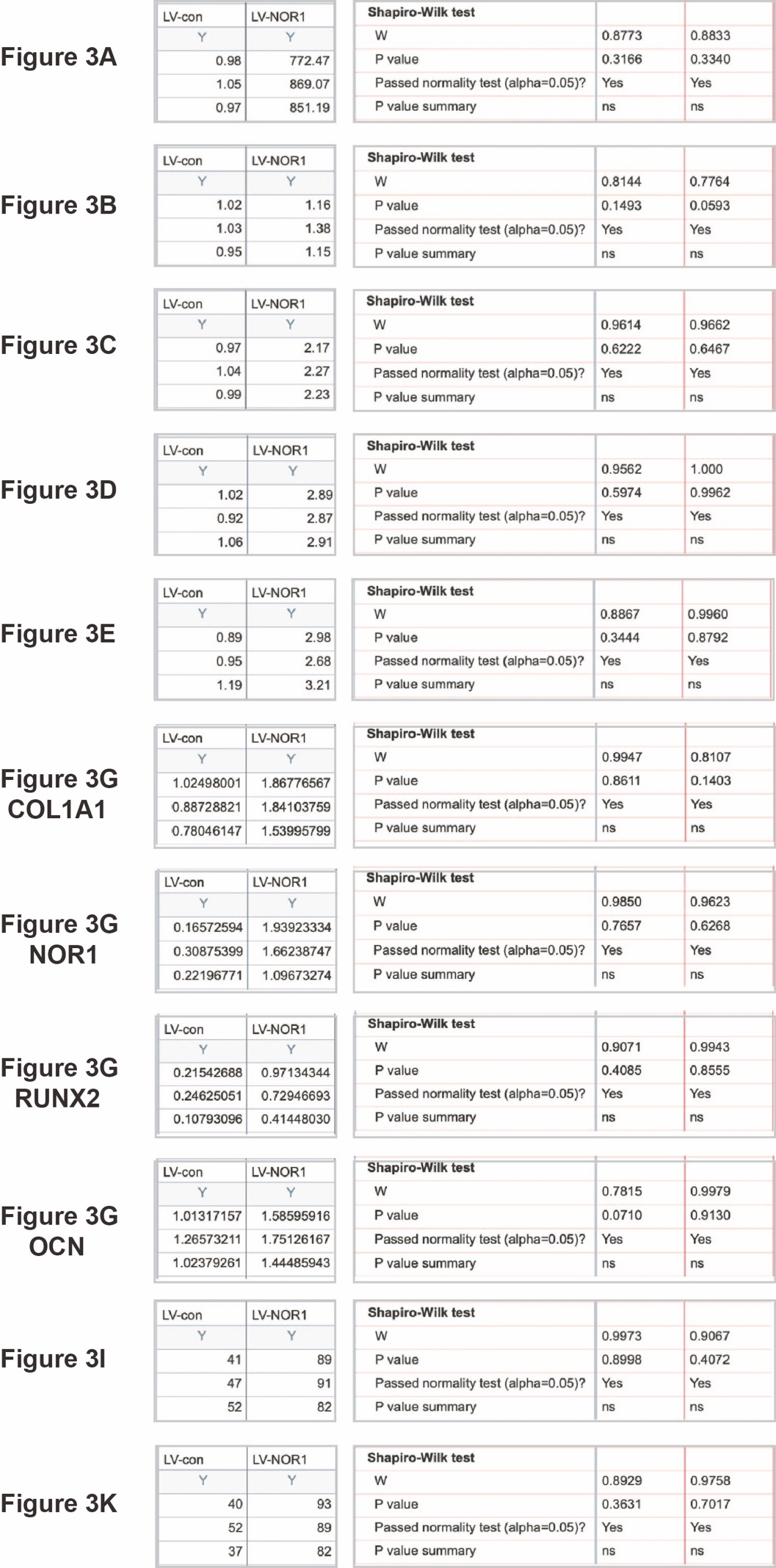


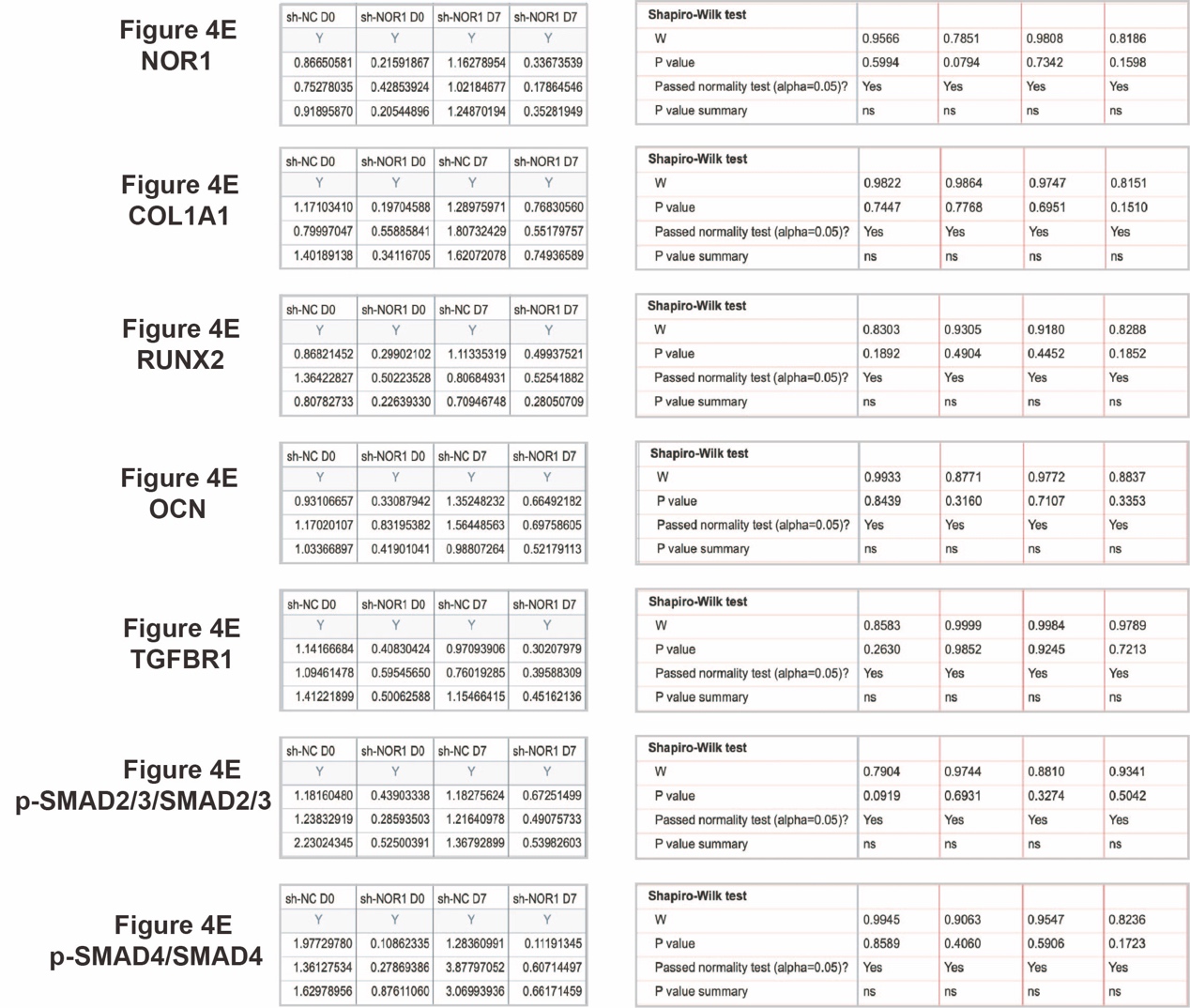


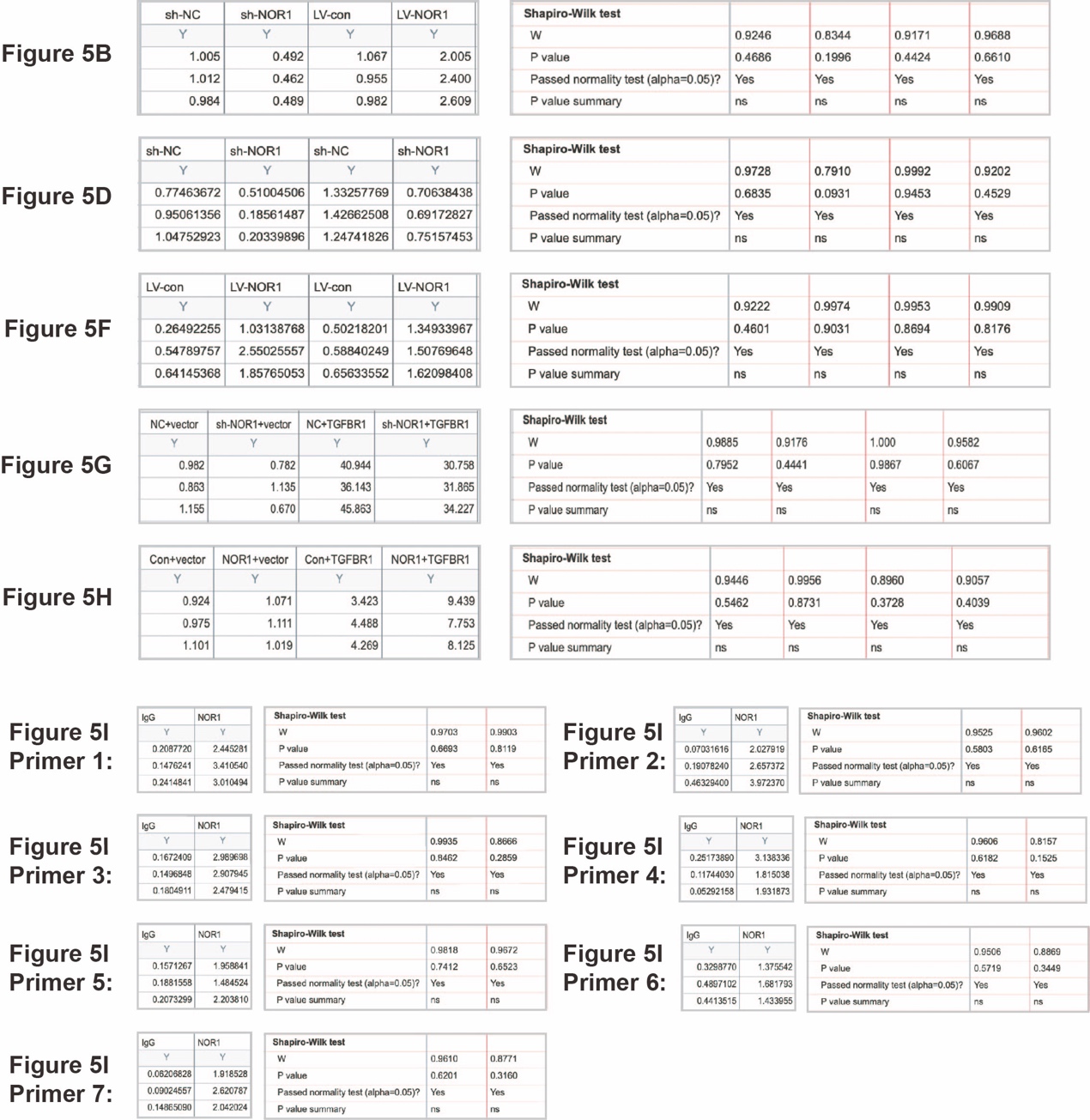


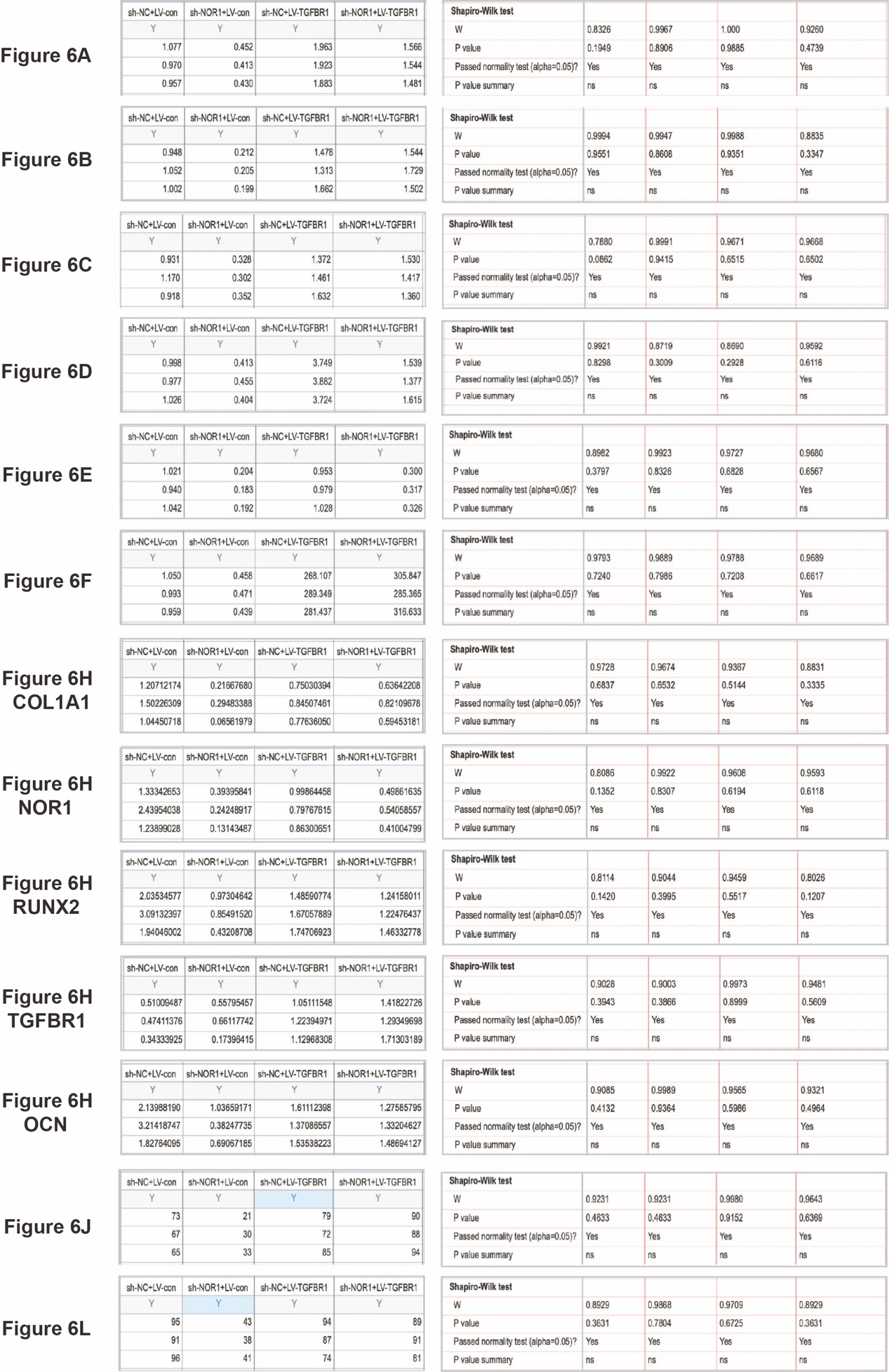


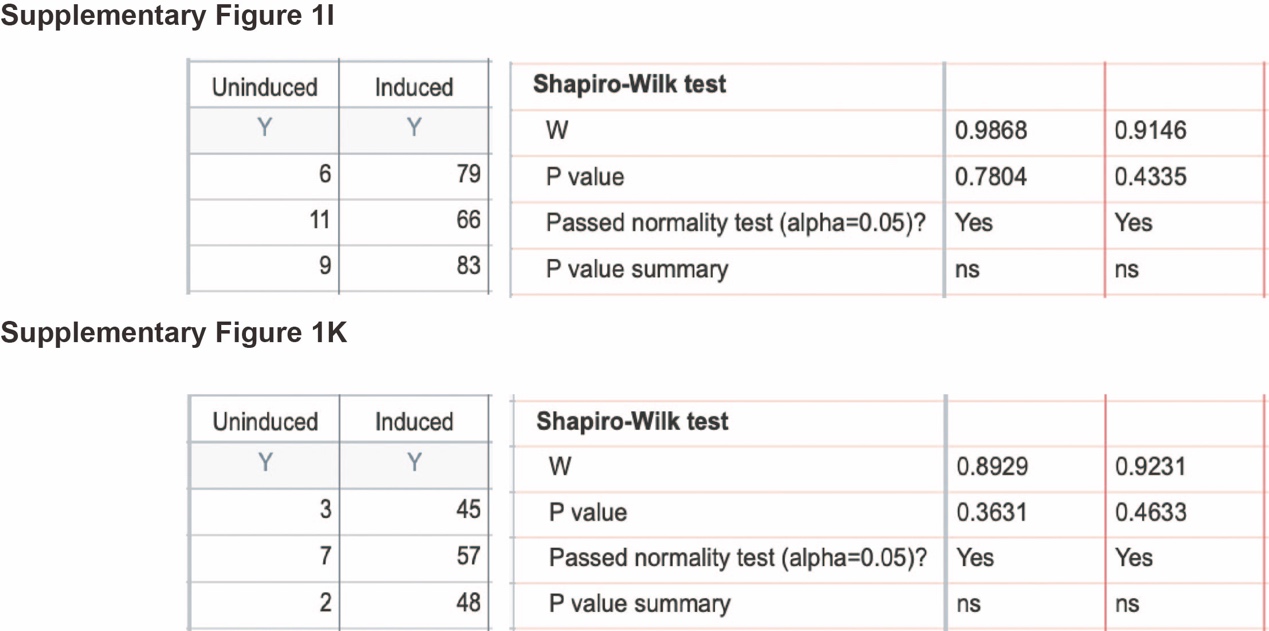


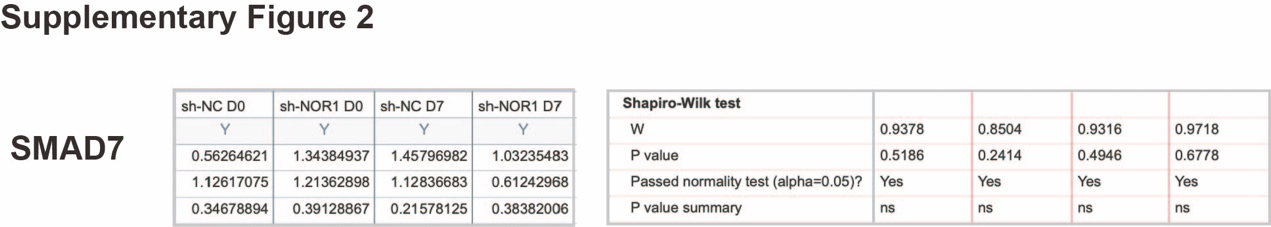

Supplement: Supplementary file 5 — Supplementary Material 5 [file 18_2024_5356_MOESM5_ESM.docx]
